# Alternative Transcripts and 3′UTR Elements Govern the Incorporation of Selenocysteine into Selenoprotein S

**DOI:** 10.1371/journal.pone.0062102

**Published:** 2013-04-16

**Authors:** Jodi L. Bubenik, Angela C. Miniard, Donna M. Driscoll

**Affiliations:** Department of Cellular and Molecular Medicine, Lerner Research Institute, Cleveland Clinic, Cleveland, Ohio, United States of America; Department of Molecular Medicine, Cleveland Clinic Lerner College of Medicine of Case Western Reserve University, Cleveland, Ohio, United States of America; German Cancer Research Center, Germany

## Abstract

Selenoprotein S (SelS) is a 189 amino acid trans-membrane protein that plays an important yet undefined role in the unfolded protein response. It has been proposed that SelS may function as a reductase, with the penultimate selenocysteine (Sec^188^) residue participating in a selenosulfide bond with cysteine (Cys^174^). Cotranslational incorporation of Sec into SelS depends on the recoding of the UGA codon, which requires a Selenocysteine Insertion Sequence (SECIS) element in the 3′UTR of the transcript. Here we identify multiple mechanisms that regulate the expression of SelS. The human SelS gene encodes two transcripts (variants 1 and 2), which differ in their 3′UTR sequences due to an alternative splicing event that removes the SECIS element from the variant 1 transcript. Both transcripts are widely expressed in human cell lines, with the SECIS-containing variant 2 mRNA being more abundant. In vitro experiments demonstrate that the variant 1 3′UTR does not allow readthrough of the UGA/Sec codon. Thus, this transcript would produce a truncated protein that does not contain Sec and cannot make the selenosulfide bond. While the variant 2 3′UTR does support Sec insertion, its activity is weak. Bioinformatic analysis revealed two highly conserved stem-loop structures, one in the proximal part of the variant 2 3′UTR and the other immediately downstream of the SECIS element. The proximal stem-loop promotes Sec insertion in the native context but not when positioned far from the UGA/Sec codon in a heterologous mRNA. In contrast, the 140 nucleotides downstream of the SECIS element inhibit Sec insertion. We also show that endogenous SelS is enriched at perinuclear speckles, in addition to its known localization in the endoplasmic reticulum. Our results suggest the expression of endogenous SelS is more complex than previously appreciated, which has implications for past and future studies on the function of this protein.

## Introduction

Selenoproteins are a diverse family of proteins characterized by the presence of selenocysteine (Sec), the 21^st^ amino acid. The incorporation of Sec into a growing peptide chain is unusual, as Sec is encoded by the UGA stop codon. Given the dual nature of this codon, specialized machinery is necessary to recode the UGA as Sec. Within the selenoprotein mRNA, a stem-loop structure called the Sec Insertion Sequence (SECIS) is required for recoding. In eukaryotes, the SECIS is found within the 3′ untranslated region (UTR) [1]. Several dedicated protein factors are also necessary for Sec insertion. SECIS-binding protein 2 (SBP2) interacts with a core motif in the SECIS element and is believed to facilitate interactions between the selenoprotein mRNA and the recoding machinery [2], [3], [4]. The binding of SBP2 to the SECIS is required for Sec insertion to occur and mutations that disrupt this interaction can lead to human disease. Many proteins are involved in the producing the Sec-tRNA^Sec^, which is non-canonical in both its synthesis and final structure [5], [6]. Sec insertion also requires a dedicated elongation factor, EFSec [7], [8] that recognizes the Sec-charged tRNA. Additional proteins have been shown to promote recoding, or to regulate synthesis of specific selenoproteins including ribosomal protein L30 [9], nucleolin [10] and eIF4a3 [11]. For a more thorough explanation, refer to reviews of selenoprotein synthesis [12], [13].

While the Sec incorporation machinery is widely expressed, the types of selenoproteins produced only partially overlap between species [14], [15]. The human selenoproteome consists of 25 family members [16]. Many selenoproteins are oxidoreductases that contain Sec at the active site. However, approximately half of the human selenoproteins are without a known function and unanticipated roles for selenoproteins are continually being discovered, as studies into the selenoproteome expand. One such example is Selenoprotein S (SelS). SelS was first identified in a screen to find genes that were differentially expressed in a diabetic animal model [17], although it was not yet recognized as a selenoprotein. It was shown to be a glucose-regulated protein, with its expression inversely proportional to circulating glucose and insulin levels [17], [18]. Recently, SelS was identified as one of the most widespread eukaryotic selenoproteins based on comparative genomics [19]. It was grouped in a protein family with Selenoprotein K, based on protein localization, domain organization and placement of Sec near the carboxy-terminus. The combination of the prevalence and conservation of SelS suggests that this protein performs an important biological function. The ability of SelS to act as a reductase was demonstrated in vitro [20], but an enzymatic activity for this protein has not been identified in cells. However, SelS was discovered to play a role in the unfolded protein response (UPR)[21]. The UPR refers to a group of conserved signaling pathways that are activated in response to the accumulation of unfolded proteins within the ER. The purpose of the UPR is to restore the ability of the ER to process its client proteins, both through the upregulation of molecular chaperones to increase folding capacity and the removal of misfolded proteins to reduce demand (ER-associated degradation, ERAD). SelS is involved in ERAD as part of a multiprotein complex that removes misfolded proteins from the ER to the cytoplasm for degradation [21]. SelS is also known as Valosin-containing protein (VCP)-Interacting Membrane Protein (VIMP) due to its interaction with VCP in this ERAD complex. The expression of SelS is upregulated under conditions of ER stress [18], presumably to help increase the capacity of a cell to manage misfolded proteins. The UPR is a crucial cellular pathway as failure to resolve ER stress will cause the cell to undergo apoptosis. Studies in multiple systems have shown that overexpression of SelS has protective effects against ER stress [22], [23], [24], while knockdown of SelS sensitizes cells to ER stress and apoptosis [22], [23], [25], [26].

Endogenously, this increase in SelS expression is facilitated by the presence of an ER-stress element (ERSE) in its promoter [27]. A naturally occurring point mutation within the ERSE of SelS led to the discovery of a second physiological function. Patients with this mutation were unable to upregulate SelS expression under ER stress conditions [28]. These patients had increased inflammation as determined by plasma levels of IL-6, IL-1β and TNF-alpha, three acute phase cytokines [28]. This inverse relationship between the expression of SelS and acute phase cytokines suggests that SelS has a role in the negative regulation of inflammation. Furthermore, siRNA knockdown of SelS in macrophage cells led to increased release of IL-6 and TNF-alpha [28], while treatment of HepG2 cells with cytokines increased SelS expression [27]. This suggests the existence of a regulatory feedback loop to control inflammatory processes. An additional line of evidence linking SelS to inflammation is its direct interaction with serum amyloid A (SAA) [17], an acute-phase inflammatory response protein, though the significance of this interaction is unknown.

ER stress and inflammation are now known to underlie many human diseases with examples that include diabetes, metabolic syndrome disorders, atherosclerosis, Alzheimer's Disease, Parkinson's Disease and non-alcoholic fatty liver disease [29], [30], [31]. Understanding the molecular mechanisms that contribute to the development and resolution of ER stress and inflammatory processes will have wide ranging contributions to human health. Given its intriguing position at the crossroads of these two processes, we were interested in investigating the expression and regulation of SelS.

In this study we show that only one of the human SelS mRNA variants can encode a selenoprotein of 189 amino acids. The other transcript encodes a truncated protein of 187 amino acids that lacks selenocysteine. Additionally, elements in the 3′UTR of the selenoprotein-encoding mRNA positively and negatively influence Sec insertion into SelS, and provide another mechanism to regulate the production of these two protein isoforms. The ability of 3′UTR elements to influence the incorporation of Sec underscores the importance of context when examining functional RNA elements such as the SECIS. We also show that in addition to being an ER-resident protein, the subcellular localization of endogenous SelS includes enrichment at perinuclear speckles adjacent to the Golgi, which was previously unknown.

## Materials and Methods

### RNA and protein sequences

All sequences were obtained using NCBI and Ensembl databases. The accession numbers for all sequences are listed in Table S1. For the RNAs, only sequences with complete 3′UTR reads were included. The presence of a SECIS element within the 3′UTR was detected with SECISearch (http://genomics.unl.edu/SECISearch.html). Most of the SelS protein sequences did not include the Sec residue. After confirming the presence of the SECIS element, the protein sequences were manually curated to include the last two residues.

### DNA constructs

Mammalian Gene Collection clones encoding SelS variant 1 (IMAGE 6450503) and SelS variant 2 (IMAGE 2967406) were purchased from Open Biosystems. The full open reading frames and 3′UTRs were cloned by PCR into the KpnI/PmeI sites of pcDNA3.1 (Invitrogen). The common SelS forward primer was 5′ GAGGGTACCGTCATGGAACGCCAAGAGG. The variant 1 reverse primer was 5′ GGCGTTTAAACGTCGTTTATTTCTA, while the variant 2 reverse primer was 5′ CGCGTTTAAACGTAATAAAAAGCTAT. The luciferase reporter construct luc/UGA^258^/PHGPx was previously described [32]. In order to generate luc/UGA^258^/SelS v1, the 3′UTR was replaced with nucleotides 649–1264 of the SelS variant 1 mRNA (NM_203472.1). The primers used to generate this product were V1luc forward 5′ CCCTTAATTAAGAATCTTGTAGAATATT and V1luc reverse 5′ CTTGCGGCCGCGTCGTTTATTTCTA. The luc/UGA^258^/SelS v2 construct includes nucleotides 649–1222 of the SelS variant 2 mRNA (NM_018445.4). All of the other luciferase constructs are derived from the SelS variant 2 mRNA. The SECIS only construct includes nucleotides 969–1090, Start-SECIS has nucleotides 649–1090, while the SECIS-end construct spans nucleotides 969–1222. The following set of primers were used in appropriate combinations to construct the v2 construct and its derivatives: V2luc forward 5′ CCCTTAATTAAGAATCTTGTTAGTGT, V2luc reverse 5′ CTTGCGGCCG CGTAATAAAAAGCTAT, SECISluc forward 5′ CCCTTAATTAAGAAATCCTTGCTGCTAGG and SECISluc reverse 5′ GAAGCGGCCGCATACAGAACAAACCCC.

The SelS constructs with V5 epitope tags used for in vitro translation/immunoprecipitation were generated by PCR amplifying the ORF of SelS without the stop codon using the common forward primer listed above and the SelS minus stop reverse primer 5′ CACTTCGAAGCCTCATCCGCCAGATGA. The PCR product was digested and subcloned into the KpnI/SfuI sites of pcDNA3.1mycHISA (Invitrogen), generating SelSmycHIS. This was subsequently digested with SfuI and AgeI and ligated with the SfuI/AgeI insert from pcDNA3.1V5His (Invitrogen), which effectively switched the epitope tag from myc to V5. The 3′UTR sequences were added between the AgeI and PmeI sites, replacing the HIS tag. Sec-V5-v2 WT contains the full-length 3′UTR, while Sec-V5-v2ΔStem removes the first 60 nucleotides of the 3′UTR. The forward primers used for generating the 3′UTR PCR products were V2-AgeI 5′CGGACCGGTTAAGAATCTTGTTAGTGT, ΔSTEM-AgeI 5′ GGCACCGGTTAAGCCTTACGCACGCTTTTC and the reverse primer was 5′ CGCGTTTAAACGTAATAAAAAGCTAT. The cysteine mutant version Cys-V5-v2 was generated using DpnI site-directed mutagenesis with the following primers: 5′ CCCGTCATCTGGCGGATGTGGCTTCGAAGGTAAGCC and 5′ GGCTTACCTTCGAAGCCACATCCGCCAGATGACGGG, where the underlined nucleotide is the altered nucleotide.

### Cell culture

HepG2 (human hepatoma), HEK293 (human embryonic kidney), and U251 (human glioma) were obtained from ATCC. All cells were cultured in a monolayer in DMEM with 1g/L glucose and 10% FBS, in 5% CO_2_ at 37°C. Cell pellets from T47D, SW480, HT29, HCT116 and HCT8 cell lines used for RNA extraction were a gift from A. Chaudhury and were all originally obtained from ATCC.

### siRNA treatment

Synthetic ON-TARGET*plus* siRNA duplexes targeting human SelS as well as non-targeting control #1 were purchased from Dharmacon. The sense sequence of the SelS siRNAs were: ACCUGAUGUUGUUGUUAAA (total SelS A), CGGAUGAGGCUAAGAAUCU (total SelS B), AGATTTACGACGTGGGAAA (variant 1-specific), and GTAAAGGCCTCTAGATGATT (variant 2-specific).

Cells were seeded in 6-well cell culture plates at 2.5×10^5^ (HEK293) or 4×10^5^ (HepG2). HEK293 treatments were performed 16 hours later with 50 nM siRNA and Dharmafect 1 transfection reagent, according to manufacturer's instructions (Dharmacon). HepG2 cells were treated with 20 nM siRNA and Dharmafect 4 transfection reagent. After 72 hours the cells were harvested for protein or were fixed for immunofluorescence (see below). Total protein lysates were obtained by washing the cells twice with phosphate buffered saline (PBS), scraping the wells, and collecting the samples in a microfuge tube. After centrifugation, the pellets were resuspended in 20 mM Tris, pH 7.5, 1% NP-40, 150 mM NaCl, 5 mM EDTA, 1 mM phenylmethylsulfonyl fluoride and HALT protease inhibitor (Pierce). The lysates were incubated for 30 minutes on ice with occasional mixing, and then centrifuged for 15 minutes at 21000 rpm in a refrigerated centrifuge. Lysates were stored at −20°C until analyzed.

### qRT-PCR

Cell pellets were obtained for each of the listed cell lines and RNA was extracted using Trizol (Invitrogen), according to manufacturer's instructions. The RNA was checked for quantity and quality using spectrophotometry and agarose gel electrophoresis. For every sample, 2 µg of RNA and random hexamer priming was used for reverse transcription using the Taqman Reverse Transcription Reagents kit (Applied Biosystems). To obtain an optimized cDNA template concentration for use in quantitative Real-Time PCR (qRT-PCR), cDNA was tested in a standard curve experiment by utilizing a10-fold dilution series over 5 points starting from the most concentrated cDNA sample. Based on these results, 2 µl of a 1∶10 dilution of cDNA template was used for qRT-PCR.

Primers in the open reading frame (ORF) were used to detect the total amount of SelS (forward: 5′-CGG TCA TGG AAC GCC AAG-3′, and reverse: 5′-GCG GAA AGC TTC TGA AAG AC-3′). Variant specific products were detected using a common forward primer in the ORF (5′-ACG GAA ATC GGA CAG AAA GC-3′) and two different reverse primers in the 3′UTRs (SelS V1: 5′-ATT TCC CTT GGT CAA GAA GCA-3′; SelS V2: 5′-GGT TCA TCT TGC TAA TGT CAA-3′). Primers for β-actin were used as a control (forward: 5′-GTC GTC GAC AAC GGC TCC GGC-3′; reverse: 5′-CCT CTC TTG CTC TGG GCC TCG-3′). For primer efficiency testing, a standard curve experiment consisting of 3 replicates of cDNA in a 10-fold dilution series using identical primer concentrations (250 nM/reaction) was performed. The primer efficiencies for each set were translated from the slope of the standard curve's linear regression line using the formula: E = (10^−1/slope^)−1.

qRT-PCR reactions were performed in triplicate with 2X Fast SYBR Green Master Mix (Applied Biosystems) and set up in MicroAmp Fast Optical 96-well reaction plates with optical caps (Applied Biosystems). Control reactions included no-reverse transcriptase controls for each cDNA template and no template controls (NTCs) for each primer set on each plate. Plates were run in a StepOnePlus Real-Time PCR System (Applied Biosystems), using conditions suggested by the Fast SYBR Green protocol (enzyme activation step: 95°C for 20 sec for 1 cycle; denature step: 95°C for 3 sec; anneal/extend step: 60°C for 30 sec; denature and anneal/extend steps repeated for 40 cycles). Data was analyzed using StepOne Software v2.1 (Applied Biosystems).

### Western blotting

Proteins were separated by SDS-PAGE and transferred to ImmunoBlot polyvinylidene fluoride (PVDF) membrane (Biorad). The primary antibodies used were α-SelS Prestige (Sigma, HPA010025), α-GAPDH (6C5) (Abcam, ab8245) and α-V5 (Life Technologies, R96025). The secondary antibodies used were either α-rabbit-HRP or α-mouse-HRP (Jackson Immunochemicals). Proteins were detected using SuperSignal West Dura Extended Duration Substrate (Thermo Scientific), and exposure to Amersham Hyperfilm ECL (GE LifeSciences). Analysis was performed using ImageQuant RT ECL (GE Healthcare).

### Luciferase-based in vitro Sec Insertion Assay

Luciferase reporter plasmid DNAs were linearized and used as templates for *in vitro* transcription using T7 RNA polymerase (Ribomax T7; Promega). *In vitro* translation reactions were assembled for a total volume of 25 µl, including 70% rabbit reticulocyte lysate (Promega), complete amino acid mixture, RNase Inhibitor and 100 ng of luc/UGA^258^ mRNA in the presence or absence of purified recombinant SBP2 CT [11]. The reactions were incubated at 30°C for 30 min. Each reaction was tested in triplicate by adding 2.5 µl of the translation mixture to 50 µl of luciferase substrate, using 5 second measurements in a 1420 Perkin Elmer Victor3 multi-label counter. The results are displayed as the mean from triplicate experiments with error bars that indicate one standard deviation, as calculated in Excel.

### V5-surrogate Sec insertion assay

The Sec-V5-v2 and Cys-V5 plasmids were linearized and used as templates for *in vitro* transcription using T7 RNA polymerase (Ribomax T7; Promega). *In vitro* translation reactions were assembled for a total volume of µl, as described above. The reactions were incubated at 30°C for 45 min. The reactions were stopped on ice, and diluted to 250 µl with 50 mM Tris-HCl, 150 mM NaCl and 1 µl of anti-SelS Prestige antibody was added to the reactions (HPA010025, SIGMA). The mixture was incubated for 1 hour at 4°C on a rotating mixer. Protein G coupled Dynabeads (Life Technologies) were used to immunoprecipitate the protein-Ab complexes. The samples were washed 3 times with 50 mM Tris-HCl, 150 mM NaCl and eluted into 2x SDS-PAGE buffer by heating to 95°C for 10 minutes. The samples were separated by SDS-PAGE and transferred to PVDF membranes. Western blotting was performed against the V5 tag using a 1∶4000 dilution of the anti-V5 antibody (R96025, Life Technologies) and a 1∶10 000 dilution of anti-MouseHRP (Jackson Immunochemicals). Blots were developed as described above.

### Immunofluorescence

Cells grown on coverslips were fixed with ice-cold methanol for 5 minutes or 4% paraformaldedye for 15 minutes. After washing with PBS, cells were permeabilized with 0.2% Triton X-100 in PBS for 5 minutes at room temperature with gentle mixing. Cells were washed twice with PBS and then incubated with ImageIt Signal Enhancer (Life Technologies) for 30 minutes. The primary antibodies were added for one hour at room temperature, washed twice in PBS and followed by incubation with the secondary antibody for one hour. After final washing, the samples were mounted onto slides using Prolong Gold Antifade with DAPI (Life Technologies) for standard immunofluorescence or Vectashield (Vector Labs) for confocal microscopy. The primary antibodies were α-SelS Prestige (Sigma, HPA010025) and α-golgin p97 clone CDF4 (Life Technologies, A21270). The secondary antibodies were Alexa Fluor 488 goat α-rabbit IgG and Alexa Fluor 568 goat α-mouse IgG (Life Technologies, A11034 and A11031, respectively). Images for standard immunofluorescence were collected on Leica DM5500B upright microscope (Leica Microsystems, GmbH) using ImagePro Plus software (MediaCybernetics). Confocal images were captured with a Leica TCS-SP2 Spectral Laser Scanning Confocal Microscope using Leica Confocal Software (Leica Microsystems, GmbH).

## Results

### SelS has two mRNA variants in humans

**Figure 1:**
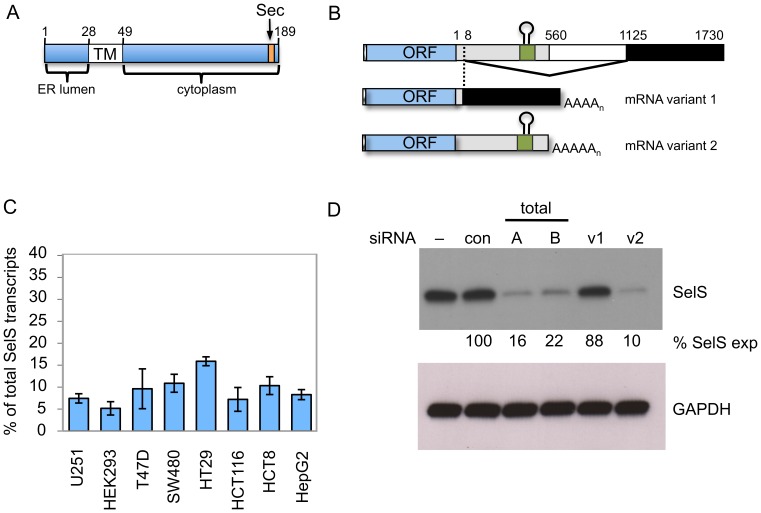
Human SelS is encoded by two variant transcripts. **A**, Schematic representation of the human SelS protein. The amino acid numbering refers to human SelS. The arrow indicates the location of the Sec residue at position 188. The ER and cytoplasmic domains are as indicated. TM, transmembrane domain. **B**, Diagram of the splicing events that generate the two variant SelS transcripts. The numbering refers to the nucleotides in the human 3′UTR sequences. The dashed line indicates the location of the 3′UTR splicing event in variant 1. The stem-loop structure indicates the location of the Selenocysteine Insertion Sequence (SECIS) element. **C**, qRT-PCR results showing the presence of the variant 1 mRNA in all cell types tested. Levels of variant 1 are expressed as a percent of the total SelS transcripts detected in the same sample. Two independent biological samples were assayed in triplicate. Results are displayed as the mean with error bars indicating one standard deviation. **D**, Representative blot from Western blot analysis of siRNA treated HEK293 cells. Cells were treated with control non-targeting siRNA (con), siRNAs that target both SelS transcripts (total A and B), or siRNAs that specifically target variant 1 (v1) or variant 2 (v2). Untreated cells were also included in the analysis (-). Total protein lysates from these cells were resolved by SDS-PAGE, transferred to PVDF and immunoblotted with a α-SelS antibody. The relative SelS protein levels were quantified and are expressed as a percent of the levels in the control lane. The same blot was reprobed for GAPDH to serve as a loading control.

SelS is a highly conserved, single-pass transmembrane protein of 189 aa that is primarily found in the endoplasmic reticulum (ER) but is also located on the cell surface. The transmembrane domain is oriented such that the small amino-terminal domain is within the ER lumen, while the larger carboxy-terminal domain is in the cytoplasm. Sec is the penultimate residue within the protein, at position 188 (Figure 1A).

Database analysis revealed that human SelS is encoded by two mRNA transcripts; variant 1 (NM_203472.1) and variant 2 (NM_018445.4). These transcripts differ in their 3′UTR sequences due to a splicing event in transcript 1 that occurs eight nucleotides into the 3′UTR (Figure 1B). Despite this difference the two transcripts are often annotated as producing the same protein, as there are no apparent alterations to their coding regions. However, the splicing event in transcript 1 excises the SECIS element, which is absolutely required for Sec insertion. Thus, these two transcripts should not be capable of producing the same protein. The variant 1 transcript would encode a 187 aa protein (without Sec), due to premature termination at the UGA codon, while the variant 2 transcript can produce the 189 aa Sec-containing protein.

We were interested in determining whether both transcripts were expressed in different cell lines. RNA samples were isolated from human cell lines derived from liver (HepG2), kidney (HEK293), colon (SW480, HT29, HCT116, HCT8), breast (T47D) and glioma (U251MG). Quantitative RT-PCR was used to examine total SelS levels using primers in the coding region, while a common forward primer and 3′UTR-specific reverse primer were used to quantify the individual variants. Each RNA sample was tested for the total SelS transcript levels, as well as the relative levels of the variant 1 and variant 2 transcripts. The SECIS-containing variant 2 transcript was predominant in all samples tested (data not shown). However, as shown in Figure 1C, the variant 1 mRNA was identified in every sample, representing 5–16% of the population of SelS transcripts across the various cell lines. In addition, the variant without the SECIS element has been detected in other primates including chimps, macaques and gibbons (Figure S1). The placement of the splice donor is preserved in other mammalian sequences (Figure S2), however there is not sufficient EST or transcriptome data to determine whether two SelS mRNA variants are expressed in other species.

We also examined the contribution of the two variants to SelS protein production using siRNA knockdown in HEK293 cells. Total SelS mRNA was targeted using two different siRNAs against the coding region of SelS, while the transcript variants were individually targeted with siRNAs designed against the 3′UTRs of each mRNA. A robust knockdown of SelS protein levels was achieved with both coding region siRNAs (80–85%), as well as the variant 2-specific siRNA (90%) when compared to treatment with a non-targeting siRNA (Figure 1D). Only a modest reduction in SelS protein was observed with the variant-1 specific siRNA (12%). These results are in good agreement with the quantitative RT-PCR results with respect to the relative abundance of the mRNA variants. Similar siRNA knockdown experiments in U251 and HepG2 cells confirmed that variant 2 is the predominant transcript in these cell lines (unpublished observations).

### Only the SelS variant 2 transcript encodes a selenoprotein

As previously mentioned, the 3′UTR of the variant 1 mRNA does not contain an identifiable SECIS element. This implies that the SelS variant 1 transcript does not encode a selenoprotein, unless a highly unusual SECIS element is present. Both transcript variants are capable of producing a SelS protein at similar levels, whether expression was examined by in vitro translation or transient transfection into cells (Figure S3). This implies that the two UTRs do not differentially effect mRNA stability or normal protein translation. As the two predicted proteins differ by 2 amino acids, they cannot be distinguished by size. We initially wished to utilize a mass spectrometry approach to discriminate between these two SelS proteins. Protein samples from untransfected cells and cells transiently transfected that overexpress SelS were examined. However, while several SelS peptides were successfully detected, the carboxy-terminal peptide was never included in the set of identified peptides, precluding mass spectrometry as a viable option. Given the technical difficulties with this approach, the ability of the two different 3′UTRs to support Sec insertion was examined using an established in vitro recoding assay. This system has been previously validated to be SECIS-dependent and codon-specific (i.e. not generalized read-through) [32]. Briefly, the assay uses a luciferase reporter construct that has been modified to contain a UGA codon at position 258, rendering expression of the luciferase protein dependent on Sec insertion (lucUGA^258^). In order to compare the ability of the two 3′UTRs to support UGA recoding, the complete 3′UTR from the SelS variant 1 mRNA (615 nucleotides) or variant 2 mRNA (573 nucleotides) was appended to the modified luciferase reporter. The SelS SECIS element (120 nucleotides) was used as a positive control. The three reporters were in vitro transcribed and translated in a rabbit reticulocyte lysate (RRL) system. Equal femtomoles of RNA were used in the reactions to account for differences in transcript size. As RRL lacks sufficient SBP2 to promote Sec incorporation, each reaction was supplemented with recombinant protein corresponding to the carboxy-terminal half of SBP2 (SBP2 CT), which contains all currently known activities of the protein and supports Sec insertion. The translation products were then assayed for luciferase activity.

**Figure 2:**
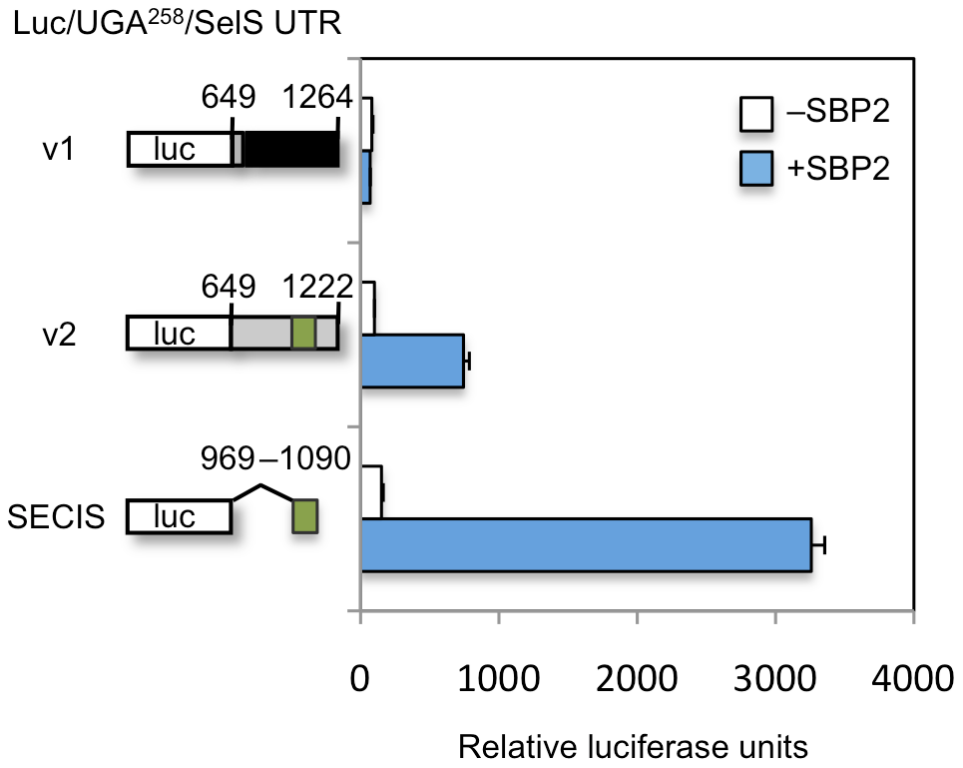
Elements in the 3′UTR inhibit SelS SECIS activity. Selenocysteine insertion activity of the two variant UTRs in vitro. The luc/UGA^258^ reporters with the variant 1 UTR, the variant 2 UTR or the SECIS only were in vitro transcribed and then translated in the presence (blue) or absence (white) of recombinant SBP2-CT. Translation products were analyzed in triplicate for luciferase activity. The results represent the mean of three separate experiments and are expressed relative to the activity of the variant 2 3′UTR in the absence of additional SBP2, which was defined as 100 relative luciferase units. The error bars represent one standard deviation. The numbering in the 3′UTRs of the constructs refers to the nucleotide numbers in GenBank sequences NM_203472 (variant 1) and NM_018445 (variant 2 and SECIS only).

In the absence of SBP2, very little luciferase is detected, which is due to low levels of non-specific readthrough. As shown in Figure 2, addition of recombinant SBP2 results in the induction of luciferase activity for the variant 2 and SECIS-only contructs (7 fold and 21 fold respectively). In contrast, the variant 1 construct does not respond to SBP2, confirming that this 3′UTR does not support UGA recoding activity. This is not due to general effects of the variant 1 3′UTR on mRNA translation as cysteine-containing versions of all the reporters were expressed at equivalent levels (data not shown). Thus, only one of the SelS mRNA variants is capable of producing a selenoprotein.

### Conserved elements in the 3′UTR of SelS

Our results show that the SelS SECIS element functions more efficiently in isolation than when found in the context of its natural 3′UTR. This suggests that other sequences are influencing the SECIS activity. We examined the sequence of the human SelS variant 2 3′UTR to look for known sequence motifs as well as potential RNA structures. Initial scanning of the sequence revealed an AU-rich region immediately downstream of the SelS SECIS element, as well as an A-rich region further downstream. No other RNA motifs were identified based on primary sequence. AU-rich elements (AREs) are well known to function in post-transcriptional gene regulation and have varied transcript-specific effects on mRNA stability and/or translational control. When SelS was used to query the AU-rich element-containing mRNA database (ARED: brp.kfshrc.edu.sa/ARED) [33], the region we identified in SelS was categorized as an ARE.

**Figure 3:**
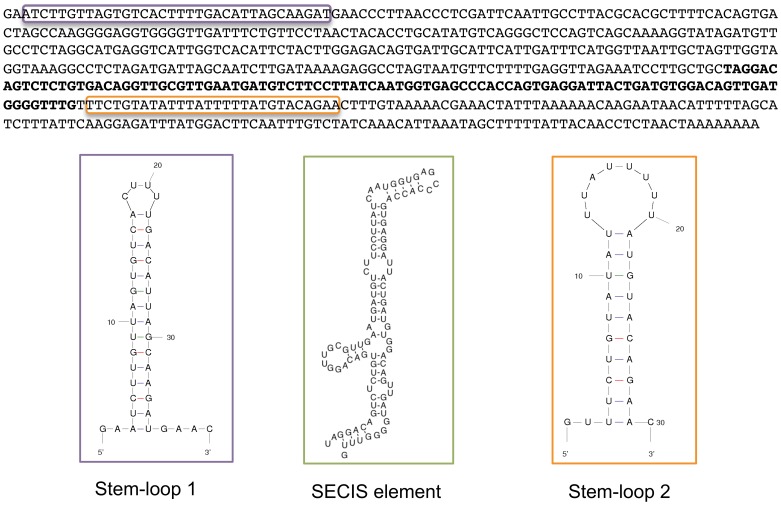
Predicted elements in the 3′UTR of human SelS variant 2 mRNA. The location of Stem-loop 1 is indicated by the purple box, while Stem-loop 2 is designated with an orange box. The SECIS element is denoted by bold font. The corresponding structural predictions are indicated for each element. The SECIS element was determined using SECISearch 2.19 (http://genome.unl.edu/SECISearch.html), while the structures for the two stem loops were predicted using RNAfold (http://rna.tbi.univie.ac.at/cgi-bin/RNAfold.cgi).

After the sequence-based searches, RNA-folding prediction programs were used to identify potential structural elements in the 3′UTR of variant 2 mRNA. First, the position of the SECIS element was determined using SECISearch 2.19 (http://genome.unl.edu/SECISearch.html) [16]. RNA-folding analysis of the entire human SelS variant 2 3′UTR using the RNAfold program from the Vienna RNA Websuite (http://rna.tbi.univie.ac.at/cgi-bin/RNAfold.cgi) [34] revealed the likelihood of two stem-loop structures within the 3′UTR (Figure 3) in addition to the SECIS element. The first stem-loop structure is located at the very beginning of the 3′UTR, and will be referred to as stem-loop 1 (SL1). This stem-loop begins three nucleotides into the 3′UTR and is situated tantalizingly close to the site of Sec insertion, in a position likely to influence recoding. The second predicted stem-loop (SL2) corresponds to the ARE identified by primary sequence analysis. This structure is predicted to form immediately downstream of the SECIS element, with the AU-rich sequence largely displayed in the loop region. AREs are often platforms for RNA-protein interactions [35]. The location of this ARE adjacent to the SECIS element makes it well placed to interfere with SECIS function depending on the complement of proteins that are present on the SECIS and the ARE, respectively.

In order to determine whether these predicted structures are conserved, a collection of available SelS sequences was assembled from NCBI and Ensembl databases. The SelS mRNA and protein sequences were obtained for as many species as possible, resulting in 32 mammalian sequences and 4 non-mammalian sequences (Table S1). Only those sequences with complete 3′UTRs were included and the presence of a SECIS element in each 3′UTR was confirmed using SECISearch. Notably, the corresponding SelS proteins were often mis-annotated in the databases, with the Sec residue absent in 22 of the 36 protein sequences. The 3′UTR sequences were then analyzed for conservation of SL1 and SL2.

**Figure 4:**
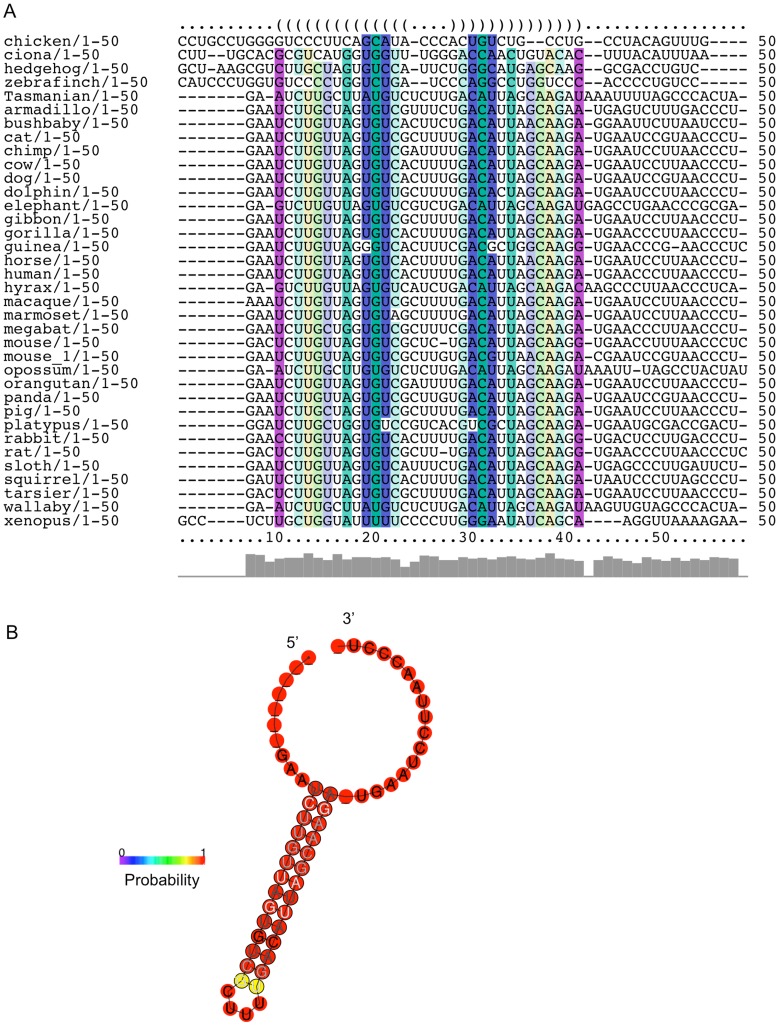
The predicted SL1 structure is conserved. **A**, The structure annotated alignment derived from the first 50 nucleotides from each SelS 3′UTR using the RNAalifold program. The color code indicates the number of base pair types found at each position: ochre-2, green-3, turquoise-4, blue-5, violet-6. Less saturated colors indicate that this base pair cannot be formed in some of the sequence set. **B**, Consensus secondary structure prediction of SL1 from RNAalifold. Nucleotides that are marked with black circles indicate locations of compensatory mutations within the sequence set. The probability of a base pair interaction is indicated on a sliding scale from 0 (blue) to 1 (red), as indicated by the legend.

Within the 3′UTR, there is very little conservation based on primary sequence outside of the SECIS element itself, even if only the mammalian sequences are analyzed. The AU-rich character of the sequence immediately downstream of the SECIS is preserved but is not identical. However, the results are different when the sequences are examined based on structural predictions instead of primary sequence. We performed an analysis of the first 50 nucleotides from each of the 3′UTRs in our collection to examine the potential structural conservation of SL1. First, the LocARNA server (http://rna.tbi.univie.ac.at/cgi-bin/LocARNA.cgi) was used to create a structural alignment of multiple RNA sequences. This output was then analyzed using the RNAalifold server (http://rna.tbi.univie.ac.at/cgi-bin/RNAalifold.cgi) to predict a consensus secondary structure for these aligned sequences. While there is some similarity across the mammalian sequences, inclusion of the non-mammalian sequences largely removes the primary sequence conservation without impacting the structural conservation of this region. Figure 4A shows the structure annotated alignment generated by the RNAalifold program [36]. The color coding of the alignment reflects the sequence covariation of this region. A mutation on one side of an RNA helix will require a matching mutation on the other side of the helix to retain the structure. Therefore, the sequence is analyzed for the six typical base pair combinations that are found in RNA helices: GC, CG, AU, UA, GU and UG. The color indicates how many of the six base pair types occur at a given position across the set of sequences. A pale version of the color denotes that not all sequences in the set can make a certain base pair. SL1 displays many examples of compensatory mutations across the predicted stem region, with several positions using multiple different base pair types. Figure 4B is the predicted consensus secondary structure for SL1 generated by the RNAalifold program. The color of the base indicates the likelihood of its involvement in a base pairing interaction. The probability scale runs from blue (low probability) to red (high probability). In addition, positions where compensatory mutations occur in the sequence set are indicated on the structure with black circles around the nucleotides. SL1 displays a high probability of forming a stem-loop structure, as the majority of the structure registers in the red range. The only exception is the base pair at the top of the stem, which likely reflects a tolerance for the helix to breathe at this position.

**Figure 5:**
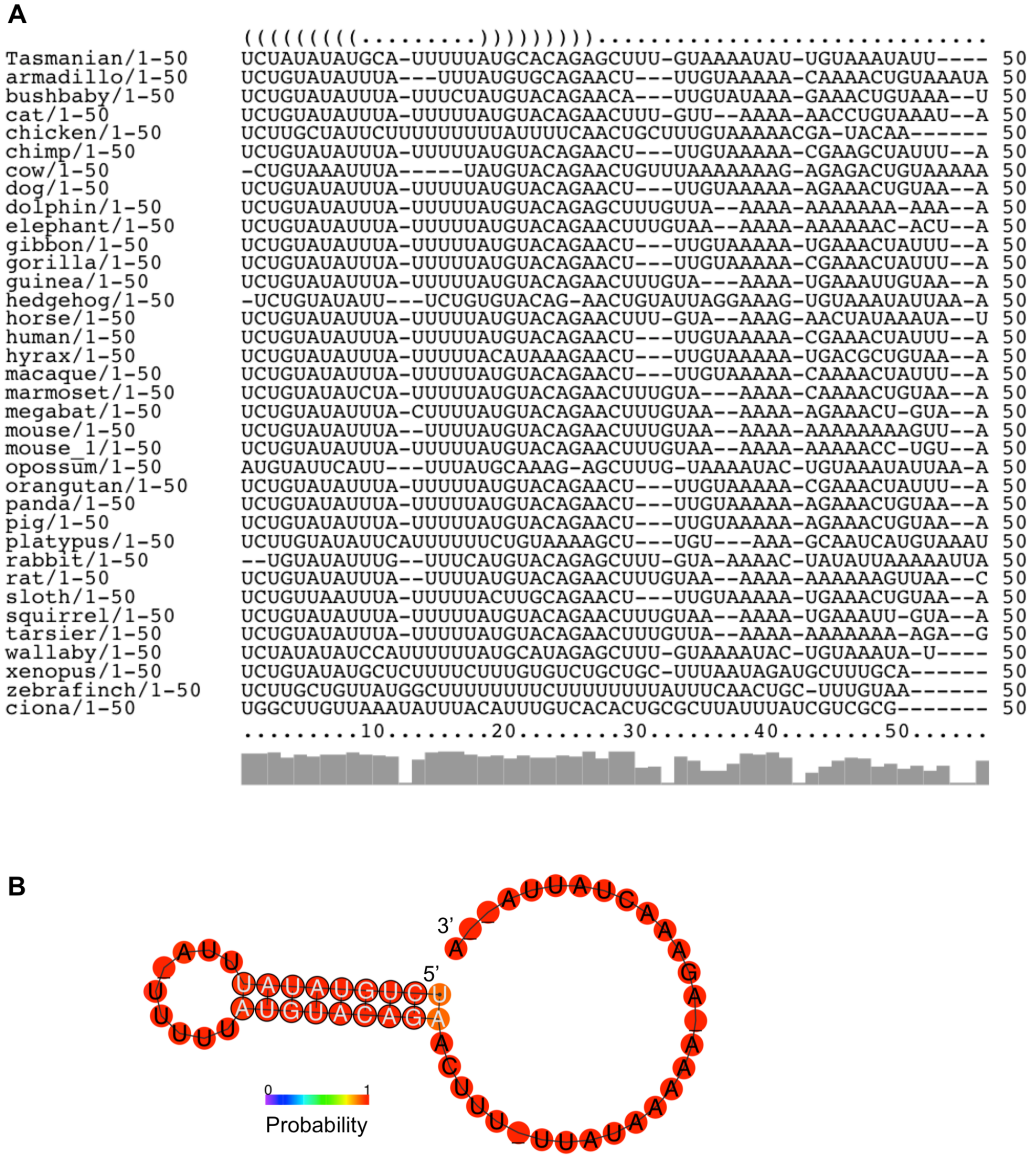
The predicted SL 2 structure is conserved. **A**, The structure annotated alignment derived from the 50 nucleotides immediately downstream of each SelS SECIS element using the RNAalifold program. **B**, Consensus secondary structure prediction of SL2 generated by RNAalifold. Nucleotides that are marked with black circles indicate locations of compensatory mutations within the sequence set. The probability of a base pair interaction is indicated on a sliding scale from 0 (blue) to 1 (red), as indicated by the legend.

For SL2, the 50 nucleotides immediately downstream from the SECIS element were used to generate the alignment. The location of the SECIS in each sequence was defined using SECISearch. Figure 5A shows the RNAalifold structure annotated sequence alignment for this region. This region of the SelS 3′UTR retains its AU-rich character across the sequence set but it is more difficult to discover sequence covariance in the region, particularly with the inclusion of non-mammalian species. Despite the sequence noise, Figure 5B shows the high-probability formation of a stem-loop structure in this region. The likelihood of the base pair interactions across the predicted stem is reinforced by the detection of compensatory mutations for each position, as indicated by black circles around the nucleotides involved. As the set of sequences is heavily weighted to mammals, we also conducted a pairwise analysis using the Ciona and Xenopus sequences in the combined locARNA/RNAalifold analysis. This analysis also predicts the formation of a stem-loop of similar size and length (data not shown). Thus, the ability to form a stem-loop structure downstream of the SECIS element is not restricted to mammals.

### Elements in the distal 3′UTR impair Sec insertion

**Figure 6:**
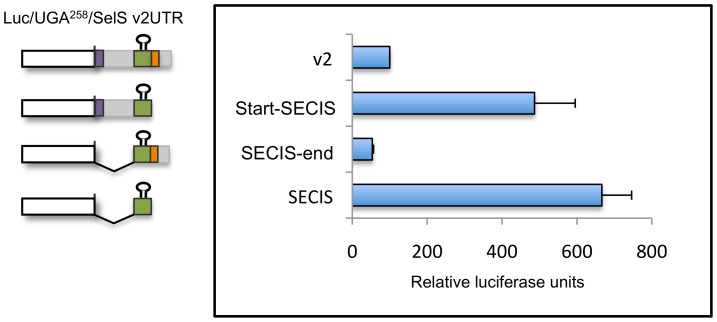
Sequences in the distal 3′UTR inhibit SECIS activity. Selenocysteine insertion activity of deletion mutants of the variant 2 3′UTR in vitro. A stem-loop structure indicates the location of the SECIS element. The luc/UGA^258^ reporter with the entire variant 2 3′UTR (v2) or portions of the 3′UTR corresponding to Start-SECIS, SECIS-end, or the SECIS only were in vitro transcribed and then translated in the presence of recombinant SBP2-CT. Translation products were analyzed in triplicate for luciferase activity. The results represent three separate experiments and are expressed relative to the activity of v2, which was defined as 100 relative luciferase units. The error bars represent one standard deviation.

Most of the studies examining Sec insertion have focused on identifying minimal SECIS elements and then examining them outside of their native context. In particular, Sec incorporation assays have often been done using minimal SECIS elements on the order of 50-200 nucleotides, but the 3′UTRs of human selenoprotein mRNAs range from 200–5000 nucleotides. Given the influence of the 3′UTR context even in this heterologous luciferase assay, we wanted to identify cis regions in the 3′UTR of SelS variant 2 that affect recoding. Therefore, we designed lucUGA^258^ constructs containing portions of the 3′UTR of SelS variant. The 3′UTR (nt 1–573) was divided into two parts based on the position of the SECIS element. The Start-SECIS construct contains nucleotides 1–441 of the UTR and ends immediately after the SECIS element. The SECIS-end construct starts just before the SECIS element and includes nucleotides 320–573 of the UTR. The complete 3′UTR (1–573) and SECIS alone (nt 320–441) were used for comparison. As shown in Figure 6, the Start-SECIS construct functions similarly to the SECIS alone. In contrast, the SECIS-end construct is severely impaired for Sec insertion, indicating that inhibitory sequences are found downstream of the SECIS element. This inhibition is not due to a change in distance of the SECIS element to the recoding event as the relative location is unchanged between the SECIS-end and SECIS only constructs.

### The ORF-proximal SL1 promotes selenocysteine insertion

While the dampening effect of the 3′UTR on the SelS SECIS is from downstream sequences, we were still interested in examining the upstream element SL1 for possible effects on Sec insertion. One could envision SL1 exerting a positive effect on Sec insertion by promoting ribosome pausing during translation. Conversely, SL1 could have a negative impact on selenoprotein synthesis by preventing the recoding machinery from accessing the UGA codon. The relative distance between this stem-loop and the UGA codon is very different in the endogenous and heterologous contexts. In its native context, SL1 is 9 nucleotides downstream of the UGA codon, whereas in the luciferase reporter there are several hundred nucleotides between them. Thus, effects caused by either steric inhibition of the Sec insertion machinery, or ribosomal pausing may not be observable in the luciferase system.

As there is no simple way to individually detect both the Sec-containing full-length SelS protein and a two amino acid truncated form, a V5 epitope tag was introduced between the UGA codon and the UAA stop codon (SelS-UGA-V5). The V5 tag is easily detectable and in these constructs the expression of the V5 tag is dependent on Sec insertion, as termination at the UGA codon would prevent inclusion of the tag.

**Figure 7:**
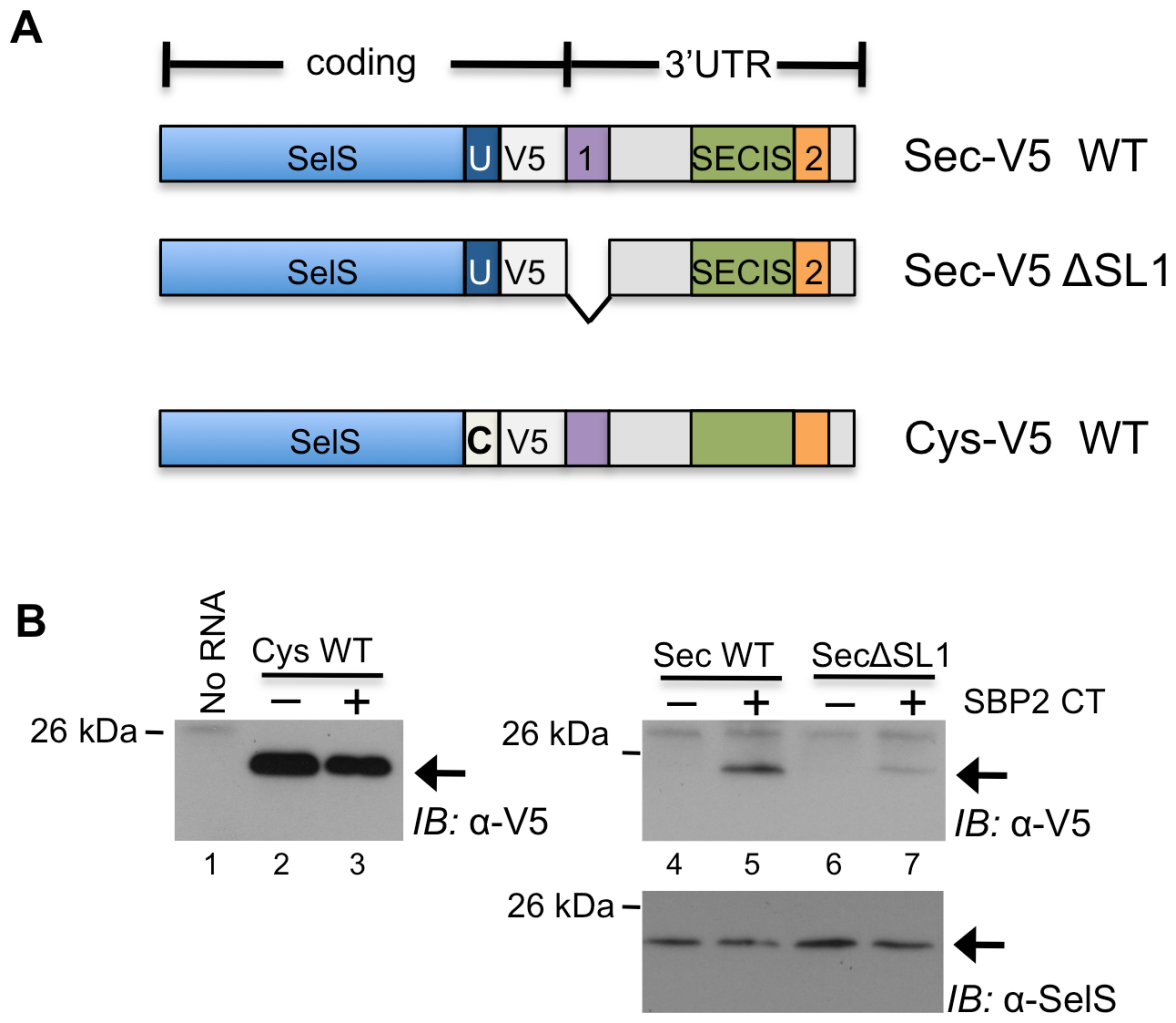
SL1 promotes Sec insertion when located in proximity to the recoding site. **A**, Schematic representation of the constructs used in this assay. The V5 epitope tag was inserted between the Sec (U) and the stop codon of the SelS open reading frame to allow detection of Sec insertion. Either the complete 3′UTR (WT) or the 3′UTR with SL1 deleted (ΔSL1) were included in the Sec constructs. A third construct that replaces the Sec (U) with a Cys (C) was included as a positive control for V5 detection in this assay. **B**&**C**, The SelS-Cys-V5 and SelS-Sec-V5 (WT and SL1) constructs were in vitro transcribed and translated, and used for immunoprecipitation (IP) against SelS. The IP reaction was resolved by SDS-PAGE and immunoblotted against the V5 epitope tag. The blot for the SelS-Sec-V5 series was stripped and reprobed for SelS. The experiment was repeated five times with similar results and a representative gel is shown.

Two SelS-UGA-V5 constructs were made that contained either the wildtype 3′UTR of SelS variant 2, or the 3′UTR with SL1 removed (Figure 7A). In addition, a third construct containing the wildtype 3′UTR was mutated to change the UGA codon to a UGU cysteine (Cys) codon (SelS-UGU-V5). The constitutive inclusion of a Cys residue instead of Sec makes the expression of the V5 tag in this construct independent of Sec insertion. This serves as a positive control for V5 expression in the assay.

The three constructs were in vitro translated using RRL in the presence or absence of SBP2 CT. As there is no detectable level of endogenous SelS in RRL, the translation products were immunoprecipitated using an anti-SelS antibody. The reactions were resolved using SDS-PAGE, transferred to PVDF membranes and immunoblotted for the V5 epitope. In order to be able to probe the samples under the same conditions, only 10% of the cysteine reaction was loaded onto the gel. As shown in Figure 7B, the SelS signal is dependent on the addition of RNA to the reactions. The SelS-UGU-V5 construct shows strong V5 signal and no dependence on SBP2-CT (left panel, lanes 2&3). As expected, both of the SelS-UGA-V5 constructs only show V5 signal in the presence of SBP2-CT. Interestingly, the removal of SL1 greatly decreases the V5 signal. This is not due a decrease in SelS production, as reprobing the membrane with an antibody directed against SelS shows that nearly equivalent SelS signals are found in both lanes (Figure 7B, compare lanes 5&7, right panel). Thus, SL1 is a positive element that appears to facilitate Sec insertion, but only when positioned in the vicinity of the recoding event.

### Endogenous SelS is found in the ER and perinuclear speckles

The above results demonstrate that the potential exists to produce two different SelS protein isoforms, a full-length protein containing a penultimate Sec residue and a truncated protein that does not contain Sec. We wondered whether the different carboxy-terminal ends would affect the subcellular localization of the protein. There are several examples where exposed thiols have been shown to be important for ER localization of proteins by mediating intramolecular bonds [37], [38], [39], [40], [41]. In addition, a precedent exists for a penultimate cysteine being required for the ER retention of the secreted immunoglobulin M heavy chain [42]. Interestingly, one study has found that SelS was secreted from HepG2 cells and appeared to be full-length based on size and the presence of an intact amino-terminal epitope tag, although the secretion of SelS was specific to HepG2 cells [43]. Given that Sec is the penultimate residue of the full-length SelS, we were interested in whether an analogous mechanism might regulate the subcellular localization for the two isoforms.

**Figure 8:**
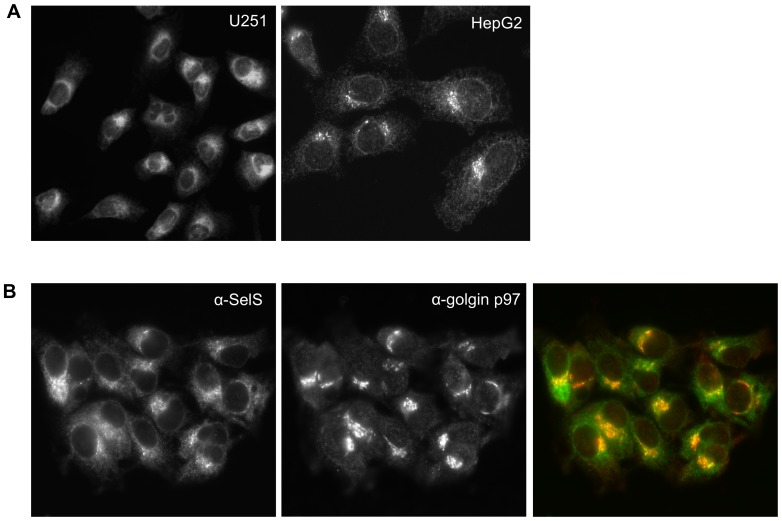
Endogenous localization of SelS includes a perinuclear accumulation. **A**, U251 cells or HepG2 cells were examined for the localization of endogenous SelS protein by immunofluorescence as described in Materials and Methods. Images were taken at 40× magnification. **B**, Costaining of SelS (green) and the Golgi (red) was performed using α-SelS and α-golgin p97 antibodies in HepG2 cells. Regions of colocalization are indicated in yellow (40× magnification).

**Figure 9:**
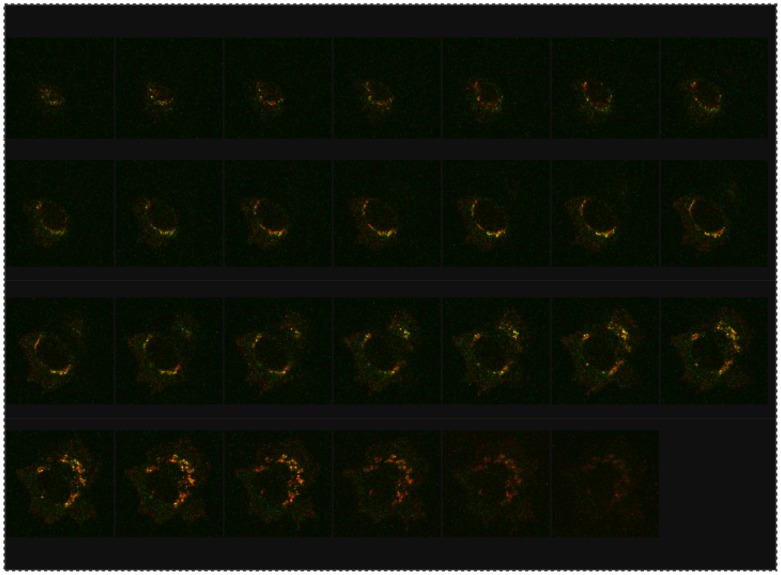
SelS localization partially overlaps with the Golgi. Confocal analysis of endogenous SelS and Golgi localization in HepG2 cells. The gallery of images depicts the z-series through a single cell. The z-axis was 9.53 µm long and each step was 0.37 µm. SelS is depicted in green, while golgin p97 is red with areas of colocalization shown in yellow.

SelS is a membrane protein and was previously shown to localize to the ER and plasma membrane by overexpression of epitope-tagged SelS constructs [21] or fractionation experiments [44]. Given the availability of a suitable antibody for immunofluorescence, we examined endogenous SelS localization. SelS is predominantly found in the ER, with some weak staining of the plasma membrane in some cells. More strikingly, there is an accumulation of SelS in a perinuclear region (Figure 8A). This localization is not cell type specific as we observed similar results in U251MG (glial) and HepG2 (liver) cells (data not shown). It is also not an artifact generated during the fixation step as acetone, methanol and 4% paraformaldehyde methods all showed this accumulation (Figure S4). Previous studies would not have observed this localization as the overexpressed SelS obscures this perinuclear signal. Given that the Golgi apparatus often shows a similar staining pattern, we concurrently stained the cells for endogenous SelS and a Golgi marker (golgin p97). As shown in Figure 8B, colocalization of these two proteins was detected next to the nucleus. In order to examine this potential colocalization more carefully, the cells were examined by confocal microscopy. A series of focal planes that spanned the depth of the cell were examined for SelS and golgin p97 localization. As shown in the image gallery, there is some spatial overlap between the two proteins, but it is not a complete colocalization (Figure 9).

**Figure 10:**
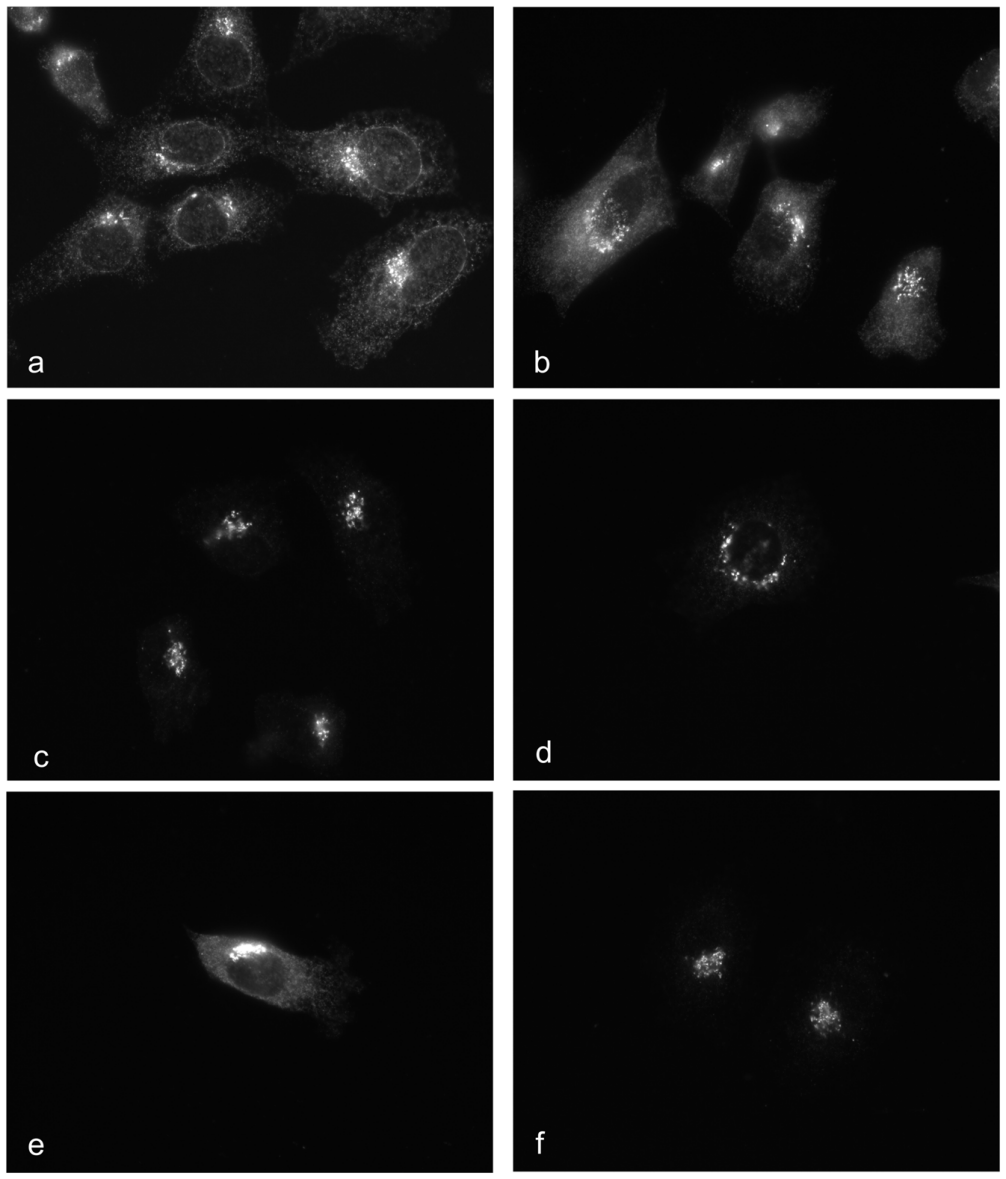
Immunofluorescence of endogenous SelS after siRNA treatment in HepG2 cells. HepG2 cells were treated with individual siRNAs as indicated. After 72 hours the cells were fixed and processed for immunofluorescence as described in Materials and Methods. Panel a: untreated cells, b: non-targeting control siRNA, c&d: siRNAs directed at the coding region that target both mRNAs, e: variant 1-specific siRNA, f: variant 2-specific siRNA.

In order to address whether these ER and perinuclear localizations might represent the two different SelS proteins (with and without Sec), we treated HepG2 cells with siRNAs directed against both SelS isoforms, as well as variant 1 and variant 2-specific siRNAs. Localization of endogenous SelS protein was examined by immunofluorescence after siRNA treatment (Figure 10). When treated with siRNAs that target both SelS mRNA variants, the punctate perinuclear signal persists, after the ER localization is no longer detectable. A similar staining pattern was observed using siRNA directed solely against the variant 2 transcript. In contrast, cells treated with the siRNA against transcript variant 1 looked similar to cells treated with a non-targeting control siRNA. Similar results were obtained with U251 cells (unpublished observation). Thus, the ER and perinuclear localizations are not simply due to two different protein isoforms from the variant mRNA transcripts. The functional significance of the perinuclear localization of the residual SelS protein is unknown but it is possible that this represents a pool of SelS protein that undergoes slower turnover than the ER population at large.

## Discussion

SelS expression has been shown to be regulated in response to cellular cues such as glucose and insulin levels, ER stress and inflammatory cytokines [17], [18], [22], [23], [27]. However, the intricacies of SelS expression have been underappreciated. In this study we identify multiple mechanisms of regulation that could affect SelS expression. First, human SelS is encoded by two variant mRNAs. Only one of the transcripts encodes a selenoprotein of 189 amino acids, while the other produces a truncated 187 amino acid protein. Additionally, cis sequences within the 3′UTR of SelS strongly influence the activity of its SECIS, providing a second mechanism to produce SelS protein isoforms with and without Sec, even in the absence of a second mRNA variant. In addition to their effects on Sec incorporation, these cis sequences may also influence other aspects of SelS mRNA metabolism through interactions with miRNAs or other RNA-binding proteins. This underscores the importance of the significance of context when studying RNA elements, such as the SECIS. While the incorporation of Sec did not affect the subcellular localization of the two protein isoforms, the loss of Sec is likely to impact protein function given that most selenoproteins are enzymes with the Sec residue at the active site. In the absence of Sec, the truncated protein may be inactive, act like a dominant negative, or have a completely different function.

It has been previously noted that there are two mRNA variants for SelS in the GenBank database. However, our study is the first to show that only one encodes a selenoprotein, contrary to the annotated comments in the database. An alternative splicing event occurs in the 3′UTR of the variant 1 transcript that removes the SECIS element, which is required for Sec insertion, while the 3′UTR of variant 2 is not spliced. Experimentally, the selenoprotein-encoding transcript is the predominant SelS mRNA, but the non-selenoprotein mRNA variant was detected in each cell line tested, representing 5–16% of the SelS transcript population. The SelS mRNA variants that lack a SECIS element have only been found in primates thus far, even though the 5′ donor splice site required for their production is conserved (Figure S2). This may be due to issues with bias or coverage in sequence sampling, or may accurately reflect the biological differences between species with respect to alternative splicing. A recent study documented that levels of alternative splicing are highest in primates [45]. In addition, alternative splicing events are more likely to be conserved between different tissues of the same species than between the same tissues of different species [45].

Even in organisms with a single SelS mRNA transcript, the ability to produce two protein isoforms of SelS remains. While many RNA elements (including SECIS elements) are often considered as independent functional units, we have identified regions within the 3′UTR of SelS mRNA that can act as positive and negative regulators of Sec insertion. First, SL1, located at the beginning of the 3′UTR, is predicted to be highly conserved across SelS sequences. We have shown that SL1 enhances Sec insertion when located proximal to the site of recoding. Previously, a similar stem-loop structure called the Sec Redefinition Element (SRE) was found 6 nucleotides downstream of the UGA codon within the coding region of Selenoprotein N [46], [47]. While a small subset of other selenoproteins are predicted to form stem-loops in locations that might act as an SRE [46], the SL1 within SelS is the second functional SRE to be identified. In addition to its function as a putative SRE, the formation of SL1 has an additional consequence in primates. RNA structures are well known to influence mRNA splicing [48]. The 5′ splice site responsible for generating the SelS variant 1 mRNA is sequestered in the double-stranded stem of SL1, preventing the splicing event. Thus, factors that influence the formation of SL1 have the potential to regulate the production of SelS variant 1 mRNAs, which cannot produce the Sec-containing SelS protein.

The 140 nucleotides region downstream of the SelS SECIS element harbors sequences that strongly inhibit Sec insertion. Within this region, one candidate is SL2, which is predicted to form immediately downstream of the SECIS element. There are different mechanisms one can envision for how the presence of this conserved element might influence Sec insertion. The presence of a stable stem-loop immediately adjacent to the SECIS element may weaken the interactions at the base of the SECIS element, interfering with its ability to form or causing destabilization. SL2 also displays an ARE, which are known to modulate transcript stability and translational control, both positively and negatively. The selenoprotein Thioredoxin reductase 1 (TrxR1) contains AREs in its 3′UTR that destabilize that mRNA [49]. However, the effects of AREs are transcript-specific, as are the protein factors that often mediate their effects [35]. The ARE in SelS does not affect the stability of the mRNA and further studies will be required to determine the mechanism by which the SelS ARE inhibits Sec insertion.

Given our findings, many of the results from previous studies on SelS need to be reinterpreted. With respect to RNA-based experiments, several studies used RT-PCR to examine SelS mRNA levels in human cell lines under various conditions [17], [18], [24], [27], [50], [51], [52], [53]. However the majority of these studies were published before the two RNA variants were annotated. Most use primer pairs in the 3′UTR of the variant 2 mRNA to examine SelS levels. In some cases this results in an underrepresentation of SelS mRNA levels. It is also not clear that both variants will respond similarly to stresses. In the case of SelS protein studies, similar caveats exist. Standard cell culture conditions are selenium deficient and hyperglycemic, which both inhibit SelS expression. Under conditions of limiting selenium, the cell prioritizes its use for the expression of essential selenoproteins, at the expense of non-essential selenoproteins, a phenomenon known as the selenoprotein hierarchy. For interpreting overexpression studies, it is often not clear that the 3′UTR or an intact SECIS element was included in the construct, which is necessary for Sec insertion. When SelS was first discovered to be a selenoprotein, it was shown that radiolabeled ^75^Se could be incorporated into a GFP-SelS fusion protein in cells, albeit relatively poorly [16]. This is likely because the SelS SECIS appears to be a poor SECIS element. In a comprehensive study that examined the minimal SECIS elements from all human selenoprotein mRNAs, the SelS SECIS was consistently among the weakest SECIS elements when tested for UGA-recoding activity in two cell lines, as well as in a cell-free system [54]. This was done using SECIS elements of ∼100 nucleotides, and our study demonstrates that the activity of the SelS SECIS is further supressed in the context of its 3′UTR. SBP2 binding is also a prerequisite for UGA recoding, and the interaction of SBP2 with the SelS SECIS is also weak [55]. In contrast, overexpression of SelS appears robust by immunofluorescence and western blot and can reach levels that distort the architecture of the ER itself [21]. The discrepancy between these observations could be explained by a mixed population of protein isoforms. Overexpression of a SelS construct that can produce a selenoprotein in cell culture would need to overcome the obstacles of a poor SECIS element, deficient selenium supply and competition for limiting SBP2 in order to be expressed in the selenoprotein form. Thus, it is likely a truncated SelS protein that does not contain Sec would be expressed under standard cell culture conditions.

**Figure 11:**
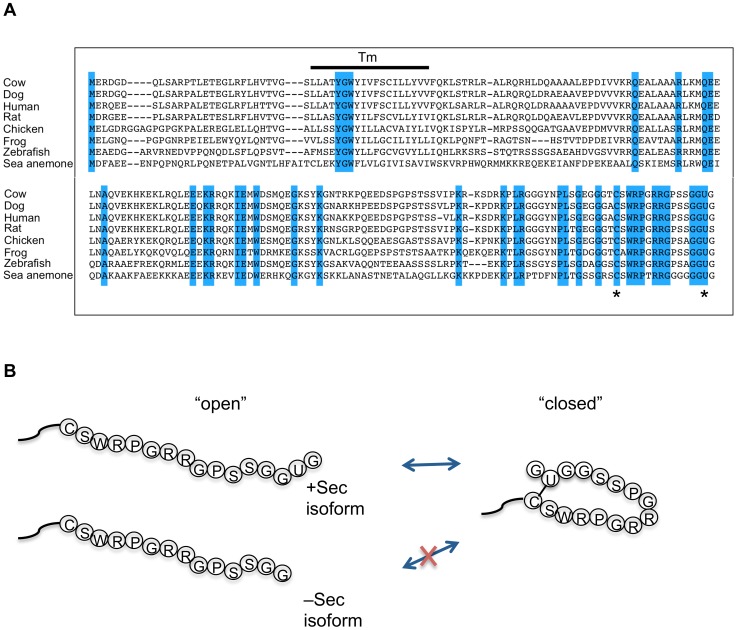
The region between the seleno-sulfide bond is conserved. **A**, Sequence alignment of SelS proteins from distantly related species demonstrates that the amino acid sequence between the reported Cys174-Sec188 bond is the most conserved region of the SelS protein. **B**, Model depicting the carboxy-terminus of SelS showing the potential effect of the truncation of SelS on regulation of the protein function.

Further support for an important role of Sec in SelS function is that the penultimate Sec participates in an intramolecular interaction that appears to affect the local conformation of the carboxy-terminus of SelS [20]. A recent study was performed to examine the structure of the cytoplasmic domain of SelS where the Sec residue was replaced by a cysteine to facilitate expression [20]. It was found that while the beginning of this protein is helical in nature, the C-terminal region is intrinsically disordered. Most interestingly, they show that a disulfide bond exists between Cys^174^ and Cys^188^, which suggests the existence of a stable selenosulfide in the native protein. Conformational changes in response to the redox state of the protein were restricted to residues 173-189. We propose that the regulation of this selenosulfide bond formation would be controlled not only in response to oxidation state, but also by the presence or absence of the Sec residue. The amino acid sequence between the Cys^174^ and Sec^188^ is also extremely conserved across SelS proteins (Figure 11A), despite the observation that disordered regions often mutate at higher rates [56], suggesting an important function for this region. It was proposed that the selenosulfide bond in SelS could function to reduce bonds in misfolded proteins that were resistant to unfolding within the ER lumen due to its higher reduction potential [20], although the conservation of the intervening sequence may reflect an additional function for this region. This suggests a model where this conserved region is caged when the selenoprotein is in its oxidized state, whereas it is flexible and available for interaction in its reduced state (Figure 11B). In contrast, the non-Sec containing protein would be constantly in an open, available state due to its inability to form a selenosulfide bond. Thus, the location of Sec within SelS may make it accessible such that its incorporation can be regulated, serving as a redox rheostat to control the function of the protein.

One quarter of all human selenoproteins share a similar placement of their Sec residue in the C-terminus (SelS, SelK, SelO, TrxR1, TrxR2 and TrxR3) [57]. Of this subset, SelS, SelO and TrxR3 are in the group of six SECIS elements identified as weak with respect to their UGA-recoding activity [54]. TrxR1 SECIS activity was on the lowest end of the moderate class. These observations make it tantalizing to wonder if Sec inclusion in this subset of selenoproteins can be regulated to control protein function. Studies on TrxR1, an essential selenoprotein that contains a penultimate Sec, reveal that the loss of Sec at the C-terminus can have profound effects on function. Substitution of the Sec residue with Cys resulted in a greatly diminished enzymatic activity, while truncation abolished its activity [49]. In cell culture, introduction of truncated TrxR1 protein without Sec results in a pro-apoptotic phenotype that is not observed with the full-length Sec-containing protein [58], [59]. This dramatic alteration in activity may underlie the finding that the cell protects against the production of truncated TrxR1 by allowing the insertion of Cys at the UGA codon under selenium deficient conditions [60], [61]. Although it cannot be ruled out that conditions may exist where the production of the truncated TrxR1 may be induced, SelS represents the first natural example of a selenoprotein with two mRNA variants where one transcript cannot produce a selenoprotein.

The information and molecular tools developed in this study will provide a strong foundation for dissecting out the functional roles for these two protein isoforms. Future studies on SelS will be directed at discriminating between Sec-dependent and independent functions and elucidating the mechanism by which sequences in the 3′UTR affect SelS function.

## Supporting Information

Figure S1 **The non-SECIS containing mRNA variant is found in multiple primates.** Clustal Omega multiple sequence alignment of the 3′UTRs from the non-SECIS containing SelS mRNA variants of macaque (ENSMMUT00000016561), chimp (GABE01007426.1), human (NM_203472.1) and gibbon (XM_003281584.2).
(DOCX)

Figure S2 **The 5**′ **splice donor site for the 3**′**UTR splicing event is conserved.** Multiple sequence alignment of the first 22 nucleotides of the 3′UTRs from the SelS mRNAs listed in Table 1. The vertical black line indicates the location of the splicing event in primates, and the canonical GT of the 5′ splice site is indicated with a horizontal black line.
(PPTX)

Figure S3 **Full-length and truncated SelS proteins are indistinguishable by size.** Western blot analysis comparing SelS proteins expressed from variant 1 and variant 2 mRNAs was performed using the α-SelS Prestige antibody (Sigma). **A**, In vitro translation reactions in RRL were programmed with in vitro transcribed RNAs for SelS-v1 or SelS-v2. A reaction without added RNA was used as a control. **B**, Transient transfection in HEK293 cells of empty vector (pcDNA3.1), SelS-v1 or Sel-v2. Arrows indicate the SelS protein products.
(PPTX)

Figure S4 **The perinuclear staining of SelS is not an artifact of the fixation method.** U251 cells were fixed either by cold acetone for 5 minutes at −20°C, cold methanol for 5 minutes at −20°C, or 4% paraformaldehyde for 15 minutes at room temperature and the effect on SelS localization was compared.
(PPTX)

Table S1 List of all accession numbers for the SECIS-containing mRNA sequences and the corresponding protein sequences used in this study. ENS-Ensembl database, NM,XM,NP,XP-Genbank database.
(DOCX)

## References

[1] Berry MJ, Banu L, Chen YY, Mandel SJ, Kieffer JD, et al (1991) Recognition of UGA as a selenocysteine codon in type I deiodinase requires sequences in the 3′ untranslated region. Nature 353: 273–276. 1832744 10.1038/353273a0

[2] Copeland PR, Fletcher JE, Carlson BA, Hatfield DL, Driscoll DM (2000) A novel RNA binding protein, SBP2, is required for the translation of mammalian selenoprotein mRNAs. EMBO J 19: 306–314. 10637234 10.1093/emboj/19.2.306 PMC305564

[3] Fletcher JE, Copeland PR, Driscoll DM, Krol A (2001) The selenocysteine incorporation machinery: interactions between the SECIS RNA and the SECIS-binding protein SBP2. RNA 7: 1442–1453. 11680849 PMC1370188

[4] Allmang C, Carbon P, Krol A (2002) The SBP2 and 15.5 kD/Snu13p proteins share the same RNA binding domain: identification of SBP2 amino acids important to SECIS RNA binding. RNA 8: 1308–1318. 12403468 10.1017/s1355838202020034 PMC1370339

[5] Lee BJ, Worland PJ, Davis JN, Stadtman TC, Hatfield DL (1989) Identification of a selenocysteyl-tRNA(Ser) in mammalian cells that recognizes the nonsense codon, UGA. J Biol Chem 264: 9724–9727. 2498338

[6] Carlson BA, Martin-Romero FJ, Kumaraswamy E, Moustafa ME, Zhi H, et al. (2001) Mammalian selenocysteine tRNA. In: Hatfield DL, editor. Selenium: Its Molecular Biology and Role in Human Health. Norwell, Massachusetts: Kluwer Academic Publishers. pp. 23–32.

[7] Tujebajeva RM, Copeland PR, Xu XM, Carlson BA, Harney JW, et al (2000) Decoding apparatus for eukaryotic selenocysteine insertion. EMBO Rep 1: 158–163. 11265756 10.1093/embo-reports/kvd033 PMC1084265

[8] Fagegaltier D, Hubert N, Yamada K, Mizutani T, Carbon P, et al (2000) Characterization of mSelB, a novel mammalian elongation factor for selenoprotein translation. EMBO J 19: 4796–4805. 10970870 10.1093/emboj/19.17.4796 PMC302067

[9] Chavatte L, Brown BA, Driscoll DM (2005) Ribosomal protein L30 is a component of the UGA-selenocysteine recoding machinery in eukaryotes. Nat Struct Mol Biol 12: 408–416. 15821744 10.1038/nsmb922

[10] Miniard AC, Middleton LM, Budiman ME, Gerber CA, Driscoll DM (2010) Nucleolin binds to a subset of selenoprotein mRNAs and regulates their expression. Nucleic Acids Res 38: 4807–4820. 20385601 10.1093/nar/gkq247 PMC2919729

[11] Budiman ME, Bubenik JL, Miniard AC, Middleton LM, Gerber CA, et al (2009) Eukaryotic initiation factor 4a3 is a selenium-regulated RNA-binding protein that selectively inhibits selenocysteine incorporation. Mol Cell 35: 479–489. 19716792 10.1016/j.molcel.2009.06.026 PMC2752292

[12] Allmang C, Wurth L, Krol A (2009) The selenium to selenoprotein pathway in eukaryotes: more molecular partners than anticipated. Biochim Biophys Acta 1790: 1415–1423. 19285539 10.1016/j.bbagen.2009.03.003

[13] Seeher S, Mahdi Y, Schweizer U (2012) Post-transcriptional control of selenoprotein biosynthesis. Curr Protein Pept Sci 13: 337–346. 22708491 10.2174/138920312801619448

[14] Mariotti M, Ridge PG, Zhang Y, Lobanov AV, Pringle TH, et al (2012) Composition and evolution of the vertebrate and mammalian selenoproteomes. PLoS One 7: e33066. 22479358 10.1371/journal.pone.0033066 PMC3316567

[15] Taskov K, Chapple C, Kryukov GV, Castellano S, Lobanov AV, et al (2005) Nematode selenoproteome: the use of the selenocysteine insertion system to decode one codon in an animal genome? Nucleic Acids Res 33: 2227–2238. 15843685 10.1093/nar/gki507 PMC1083425

[16] Kryukov GV, Castellano S, Novoselov SV, Lobanov AV, Zehtab O, et al (2003) Characterization of mammalian selenoproteomes. Science 300: 1439–1443. 12775843 10.1126/science.1083516

[17] Walder K, Kantham L, McMillan JS, Trevaskis J, Kerr L, et al (2002) Tanis: a link between type 2 diabetes and inflammation? Diabetes 51: 1859–1866. 12031974 10.2337/diabetes.51.6.1859

[18] Gao Y, Feng HC, Walder K, Bolton K, Sunderland T, et al (2004) Regulation of the selenoprotein SelS by glucose deprivation and endoplasmic reticulum stress - SelS is a novel glucose-regulated protein. FEBS Lett 563: 185–190. 15063746 10.1016/S0014-5793(04)00296-0

[19] Shchedrina VA, Everley RA, Zhang Y, Gygi SP, Hatfield DL, et al (2011) Selenoprotein K binds multiprotein complexes and is involved in the regulation of endoplasmic reticulum homeostasis. J Biol Chem 286: 42937–42948. 22016385 10.1074/jbc.M111.310920 PMC3234841

[20] Christensen LC, Jensen NW, Vala A, Kamarauskaite J, Johansson L, et al (2012) The human selenoprotein VCP-interacting membrane protein (VIMP) is non-globular and harbors a reductase function in an intrinsically disordered region. J Biol Chem 287: 26388–26399. 22700979 10.1074/jbc.M112.346775 PMC3406722

[21] Ye Y, Shibata Y, Yun C, Ron D, Rapoport TA (2004) A membrane protein complex mediates retro-translocation from the ER lumen into the cytosol. Nature 429: 841–847. 15215856 10.1038/nature02656

[22] Kim KH, Gao Y, Walder K, Collier GR, Skelton J, et al (2007) SEPS1 protects RAW264.7 cells from pharmacological ER stress agent-induced apoptosis. Biochem Biophys Res Commun 354: 127–132. 17210132 10.1016/j.bbrc.2006.12.183 PMC1855283

[23] Fradejas N, Serrano-Perez Mdel C, Tranque P, Calvo S (2011) Selenoprotein S expression in reactive astrocytes following brain injury. Glia 59: 959–972. 21456042 10.1002/glia.21168

[24] Kelly E, Greene CM, Carroll TP, McElvaney NG, O'Neill SJ (2009) Selenoprotein S/SEPS1 modifies endoplasmic reticulum stress in Z variant alpha1-antitrypsin deficiency. J Biol Chem 284: 16891–16897. 19398551 10.1074/jbc.M109.006288 PMC2719325

[25] Fradejas N, Pastor MD, Mora-Lee S, Tranque P, Calvo S (2008) SEPS1 gene is activated during astrocyte ischemia and shows prominent antiapoptotic effects. J Mol Neurosci 35: 259–265. 18498015 10.1007/s12031-008-9069-3

[26] Du S, Liu H, Huang K (2010) Influence of SelS gene silence on beta-Mercaptoethanol-mediated endoplasmic reticulum stress and cell apoptosis in HepG2 cells. Biochim Biophys Acta 1800: 511–517. 20114070 10.1016/j.bbagen.2010.01.005

[27] Gao Y, Hannan NR, Wanyonyi S, Konstantopolous N, Pagnon J, et al (2006) Activation of the selenoprotein SEPS1 gene expression by pro-inflammatory cytokines in HepG2 cells. Cytokine 33: 246–251. 16574427 10.1016/j.cyto.2006.02.005

[28] Curran JE, Jowett JB, Elliott KS, Gao Y, Gluschenko K, et al (2005) Genetic variation in selenoprotein S influences inflammatory response. Nat Genet 37: 1234–1241. 16227999 10.1038/ng1655

[29] Yoshida H (2007) ER stress and diseases. FEBS J 274: 630–658. 17288551 10.1111/j.1742-4658.2007.05639.x

[30] Hotamisligil GS (2010) Endoplasmic reticulum stress and the inflammatory basis of metabolic disease. Cell 140: 900–917. 20303879 10.1016/j.cell.2010.02.034 PMC2887297

[31] Wang S, Kaufman RJ (2012) The impact of the unfolded protein response on human disease. J Cell Biol 197: 857–867. 22733998 10.1083/jcb.201110131 PMC3384412

[32] Mehta A, Rebsch CM, Kinzy SA, Fletcher JE, Copeland PR (2004) Efficiency of mammalian selenocysteine incorporation. J Biol Chem 279: 37852–37859. 15229221 10.1074/jbc.M404639200 PMC2820281

[33] Bakheet T, Williams BR, Khabar KS (2006) ARED 3.0: the large and diverse AU-rich transcriptome. Nucleic Acids Res 34: D111–114. 16381826 10.1093/nar/gkj052 PMC1347415

[34] Gruber AR, Lorenz R, Bernhart SH, Neubock R, Hofacker IL (2008) The Vienna RNA websuite. Nucleic Acids Res 36: W70–74. 18424795 10.1093/nar/gkn188 PMC2447809

[35] Barreau C, Paillard L, Osborne HB (2005) AU-rich elements and associated factors: are there unifying principles? Nucleic Acids Res 33: 7138–7150. 16391004 10.1093/nar/gki1012 PMC1325018

[36] Hofacker IL (2007) RNA consensus structure prediction with RNAalifold. Methods Mol Biol 395: 527–544. 17993696 10.1007/978-1-59745-514-5_33

[37] Kerem A, Kronman C, Bar-Nun S, Shafferman A, Velan B (1993) Interrelations between assembly and secretion of recombinant human acetylcholinesterase. J Biol Chem 268: 180–184. 8416926

[38] Fra AM, Fagioli C, Finazzi D, Sitia R, Alberini CM (1993) Quality control of ER synthesized proteins: an exposed thiol group as a three-way switch mediating assembly, retention and degradation. EMBO J 12: 4755–4761. 8223484 10.1002/j.1460-2075.1993.tb06164.x PMC413922

[39] Guenzi S, Fra AM, Sparvoli A, Bet P, Rocco M, et al (1994) The efficiency of cysteine-mediated intracellular retention determines the differential fate of secretory IgA and IgM in B and plasma cells. Eur J Immunol 24: 2477–2482. 7925577 10.1002/eji.1830241033

[40] Reddy P, Sparvoli A, Fagioli C, Fassina G, Sitia R (1996) Formation of reversible disulfide bonds with the protein matrix of the endoplasmic reticulum correlates with the retention of unassembled Ig light chains. EMBO J 15: 2077–2085. 8641273 PMC450129

[41] Otsu M, Bertoli G, Fagioli C, Guerini-Rocco E, Nerini-Molteni S, et al (2006) Dynamic retention of Ero1alpha and Ero1beta in the endoplasmic reticulum by interactions with PDI and ERp44. Antioxid Redox Signal 8: 274–282. 16677073 10.1089/ars.2006.8.274

[42] Sitia R, Neuberger M, Alberini C, Bet P, Fra A, et al (1990) Developmental regulation of IgM secretion: the role of the carboxy-terminal cysteine. Cell 60: 781–790. 2107027 10.1016/0092-8674(90)90092-s

[43] Gao Y, Pagnon J, Feng HC, Konstantopolous N, Jowett JB, et al (2007) Secretion of the glucose-regulated selenoprotein SEPS1 from hepatoma cells. Biochem Biophys Res Commun 356: 636–641. 17374524 10.1016/j.bbrc.2007.03.018

[44] Gao Y, Walder K, Sunderland T, Kantham L, Feng HC, et al (2003) Elevation in Tanis expression alters glucose metabolism and insulin sensitivity in H4IIE cells. Diabetes 52: 929–934. 12663463 10.2337/diabetes.52.4.929

[45] Barbosa-Morais NL, Irimia M, Pan Q, Xiong HY, Gueroussov S, et al (2012) The evolutionary landscape of alternative splicing in vertebrate species. Science 338: 1587–1593. 23258890 10.1126/science.1230612

[46] Howard MT, Aggarwal G, Anderson CB, Khatri S, Flanigan KM, et al (2005) Recoding elements located adjacent to a subset of eukaryal selenocysteine-specifying UGA codons. EMBO J 24: 1596–1607. 15791204 10.1038/sj.emboj.7600642 PMC1142574

[47] Howard MT, Moyle MW, Aggarwal G, Carlson BA, Anderson CB (2007) A recoding element that stimulates decoding of UGA codons by Sec tRNA[Ser]Sec. RNA 13: 912–920. 17456565 10.1261/rna.473907 PMC1869034

[48] Warf MB, Berglund JA (2010) Role of RNA structure in regulating pre-mRNA splicing. Trends Biochem Sci 35: 169–178. 19959365 10.1016/j.tibs.2009.10.004 PMC2834840

[49] Gasdaska JR, Harney JW, Gasdaska PY, Powis G, Berry MJ (1999) Regulation of human thioredoxin reductase expression and activity by 3′-untranslated region selenocysteine insertion sequence and mRNA instability elements. J Biol Chem 274: 25379–25385. 10464265 10.1074/jbc.274.36.25379

[50] Karlsson HK, Tsuchida H, Lake S, Koistinen HA, Krook A (2004) Relationship between serum amyloid A level and Tanis/SelS mRNA expression in skeletal muscle and adipose tissue from healthy and type 2 diabetic subjects. Diabetes 53: 1424–1428. 15161744 10.2337/diabetes.53.6.1424

[51] Seiderer J, Dambacher J, Kuhnlein B, Pfennig S, Konrad A, et al (2007) The role of the selenoprotein S (SELS) gene -105G>A promoter polymorphism in inflammatory bowel disease and regulation of SELS gene expression in intestinal inflammation. Tissue Antigens 70: 238–246. 17661913 10.1111/j.1399-0039.2007.00888.x

[52] Du JL, Sun CK, Lu B, Men LL, Yao JJ, et al (2008) Association of SelS mRNA expression in omental adipose tissue with Homa-IR and serum amyloid A in patients with type 2 diabetes mellitus. Chin Med J (Engl) 121: 1165–1168. 18710632

[53] Zeng J, Du S, Zhou J, Huang K (2008) Role of SelS in lipopolysaccharide-induced inflammatory response in hepatoma HepG2 cells. Arch Biochem Biophys 478: 1–6. 18675776 10.1016/j.abb.2008.07.016

[54] Latreche L, Jean-Jean O, Driscoll DM, Chavatte L (2009) Novel structural determinants in human SECIS elements modulate the translational recoding of UGA as selenocysteine. Nucleic Acids Res 37: 5868–5880. 19651878 10.1093/nar/gkp635 PMC2761289

[55] Donovan J, Copeland PR (2012) Selenocysteine insertion sequence binding protein 2L is implicated as a novel post-transcriptional regulator of selenoprotein expression. PLoS One 7: e35581. 22530054 10.1371/journal.pone.0035581 PMC3328465

[56] Brown CJ, Johnson AK, Dunker AK, Daughdrill GW (2011) Evolution and disorder. Curr Opin Struct Biol 21: 441–446. 21482101 10.1016/j.sbi.2011.02.005 PMC3112239

[57] Driscoll DM, Chavatte L (2004) Finding needles in a haystack. In silico identification of eukaryotic selenoprotein genes. EMBO Rep 5: 140–141. 14755306 10.1038/sj.embor.7400080 PMC1298991

[58] Anestal K, Arner ES (2003) Rapid induction of cell death by selenium-compromised thioredoxin reductase 1 but not by the fully active enzyme containing selenocysteine. J Biol Chem 278: 15966–15972. 12574159 10.1074/jbc.M210733200

[59] Anestal K, Prast-Nielsen S, Cenas N, Arner ES (2008) Cell death by SecTRAPs: thioredoxin reductase as a prooxidant killer of cells. PLoS One 3: e1846. 18382651 10.1371/journal.pone.0001846 PMC2268967

[60] Lu J, Zhong L, Lonn ME, Burk RF, Hill KE, et al (2009) Penultimate selenocysteine residue replaced by cysteine in thioredoxin reductase from selenium-deficient rat liver. FASEB J 23: 2394–2402. 19351701 10.1096/fj.08-127662 PMC2717770

[61] Xu XM, Turanov AA, Carlson BA, Yoo MH, Everley RA, et al (2010) Targeted insertion of cysteine by decoding UGA codons with mammalian selenocysteine machinery. Proc Natl Acad Sci U S A 107: 21430–21434. 21115847 10.1073/pnas.1009947107 PMC3003055

