# Supplementary material for: Alternative Transcripts and 3′UTR Elements Govern the Incorporation of Selenocysteine into Selenoprotein S
Source: PLoS One. 2013 Apr 16;8(4):e62102. doi: 10.1371/journal.pone.0062102 (PMC3628699; doi:10.1371/journal.pone.0062102)
Supplement: Figure S1 — The non-SECIS containing mRNA variant is found in multiple primates. Clustal Omega multiple sequence alignment of the 3′UTRs from the non-SECIS containing SelS mRNA variants of macaque (ENSMMUT00000016561), chimp (GABE01007426.1), human (NM_203472.1) and gibbon (XM_003281584.2). (DOCX) [file pone.0062102.s001.docx]

macaque AAATCTTGTAGAATATTCCGTGGTTGCTTCTTGACAGAGGGAAACACAGTCTGGCCGTGC

chimp GAATCTTGTAGAATATTCCGTGGTTGCTTCTTGACCAAGGGAAATACAGTCTGGCTGTGC

human GAATCTTGTAGAATATTCCGTGGTTGCTTCTTGACCAAGGGAAATACAGTCTGGCTGTGA

gibbon GAATCTTGTAGAATATTCCGTGGTTGCTTCTTGACCAAGGGAAATACAGTCTGGCTGTGC

********************************** ******* ********** ***

macaque GAGACTTAAAATCTCTTGAGGAGCGCTCTGGAGAATGGCTGAAGGAGAGAAAACAGGAGC

chimp GAGACTTAAAATCTCTTGAGGAGCGCTCTGGAGAATGGCTGAAGGAGAGGAAACAGGAGC

human GAGACTTAAAATCTCTTGAGGAGCGCTCTGGAGAATGGCTGAAGGAGAGGAAACAGGAGC

gibbon GAGACTTAAAA--TCTTGAGGAGCGCTCTGGAGAGTGGCTGGAGGAGAGGAAACAGGAGC

*********** ********************* ****** ******* **********

macaque CTTGAGCAGTGTAATTACAAACAAATAGGTTGGCATAGTCAAGACTAAGTCTGTGAGTCT

chimp CTTGAGCAGTGTAATTACAAACAAATAGGTTGGCATAGTC----TTAAGTCTTTGAGTCT

human CTTGAGCAGTGTAATTACAAACAAATAGGTTGGCATAGTC----TTAAGTCTTTGAGTCT

gibbon CTTGGGCAGCGTAATTACAAACAAATAGGTTGGCATAGTC----TTAAGTCTTTGAGTCT

**** **** ****************************** ******* *******

macaque AGAGAGACTTGAGTTTTATTTTGCCTGTGGAGGAAATTGGGGGTTTCAGGTCACAGAGAG

chimp AGAGAGATTTGAGTTTTGTTTTGCCTGTGGAGGAAATTGGGGGTTTCAGGTCAAAGAGAG

human AGAGAGATTTGAGTTTTGTTTTGCCTGTGGAGGCAATTGGGGGTTTCAGGTCAAAGAGAG

gibbon AGAGAGATTTGAGTTTTATTTTGCCTGTGGAGGAAATTGGGGGTTTCAGGTCAAAGAGAG

******* ********* *************** ******************* ******

macaque GGTCAGTGGAAACAAGGGTGGACCTTGTGAGAGGTGTGGGGAAGTCCTGGGACCCTCACT

chimp GGTCAGTGGAAACAAGGGTGGGCCTTGT--GAGGTGTGGGGAAGCCCTGGGACCCTCACT

human GGTCAGTGGAAACAAGGGTGGGCCTTGT--GAGGTGTGGGGAAGCCCTGGGACCCTCACT

gibbon GGTCGGTGGAAACAAGGGTGGGCCTTGT--GAGGTGTGGGGAAGCCCTGGGACCCTCACT

**** **************** ****** ************** ***************

macaque CCCCTTCCAGTGTGTAACTGGATTGGCTCCCACCAGCACAGAAGATTTATGATGTGGGAA

chimp CCCCTTCCGGTGTGTAACTGGATT-GCTCCCACCAGCACAGAAGATCTACGACGTGGGAA

human CCCCTTCCAGTGTGTAACTGGATTGGCTCCCACCAGCCCAGAAGATTTACGACGTGGGAA

gibbon CCCCTTCCAGTGTGTAACTGGATTGGCTCCCACCAGCACAGAAGATTGACGACATGGGAA

******** *************** ************ ******** * ** ******

macaque ATGGTATACCTGGATTAAAATATTTCATCCAGATTTGTTTACATCTAGAGAGAACCCCTT

chimp ATGGTATACTTGGATTAAAATATTTCATCCAGATTTGTTTACATCTAGAGAGAACCCCTT

human ATGGTATACTTGGATTAAAATATTTCATCCAGATTTGTTTACATCTAGAGAGAACCCCTT

gibbon ATTGTATACTCGGATTACAATATTTCATCCAGATTTGTTTACAGCTAGAGAGAACCCCTT

** ****** ****** ************************* ****************

macaque GTAGGATATAAGGGAACTTTTTAACATTCTTCCTTGAGCATACTTTCTGTAACTGAAAAT

chimp GTAGGTTATAAGGAAACTTTTTAACATTCTTCCTTGAATATATTTTCTGTAGCTGAAAAT

human GTAGGTTATAAGGAAACTTTTTAACATTCTTCCTTGAATATATTTTCTGTAGCTGAAAAT

gibbon GTAGGATATAAGGAAAC--TTTAACATTCTTCCTTGAATATATTTTCTGTAGCTGAAAAT

***** ******* *** ****************** *** ******** ********

macaque GTATGTGAAGTGGCTGTCAACTACACATTTCATAAGTAGAGGGGCTCTGGGTCAGGGTTT

chimp GTTTGTGAAGTGGCTGTCAACTACACATTGCATAAGGAGAGGGGCTCTGGGTCGGGGTTT

human GTTTGTGAAGTGGCTGTCAACTACACATCGCATGAGTAGAGGGGCTCTGGGTCGGGGTTT

gibbon GTATGTGAAGTGGCTGCCAACTACACATTTCATAAGTAGAGGGGCTCTGGGTCAGGGTTT

** ************* *********** *** ** **************** ******

macaque CCTATGCAGCAGAGCAGCCCCCTTGCTGCCGTCCATCAAGAGCC----------------

chimp CATATGCAGCAGAGCAGCTCCCTTGCTGCCGTCCATCAAGAGCCCTCAACAC--GAGTTT

human CATATGCAGCAGAGCAGCTCCCTTGCTGCCGTCCATCAAGAGCCCTCAACACAAGAGTTT

gibbon CATATACAGCAGAGCAGTTCCCTCGCTGCCGTCCATCAAGAGCCCTCAACACAAGAGTTT

* *** *********** **** ********************

macaque ---------------------------------------------------

chimp GTTATAAATAGAAATAAAAGACAAAAAGTAGAGGGGTATGTTTAAGTTCAC

human GTTATAAATAGAAATAAACGACAAAAAGTAGAGGGGTATGTTTAAGTACA-

gibbon GTTATAAATAGAAATAAACTACGAAAAGTAGA-------------------

Sup Fig 1
