# Supplementary material for: Alternative Transcripts and 3′UTR Elements Govern the Incorporation of Selenocysteine into Selenoprotein S
Source: PLoS One. 2013 Apr 16;8(4):e62102. doi: 10.1371/journal.pone.0062102 (PMC3628699; doi:10.1371/journal.pone.0062102)
Supplement: Figure S2 — The 5′ splice donor site for the 3′UTR splicing event is conserved. Multiple sequence alignment of the first 22 nucleotides of the 3′UTRs from the SelS mRNAs listed in Table 1. The vertical black line indicates the location of the splicing event in primates, and the canonical GT of the 5′ splice site is indicated with a horizontal black line. (PPTX) [file pone.0062102.s002.pptx]

## Slide 1
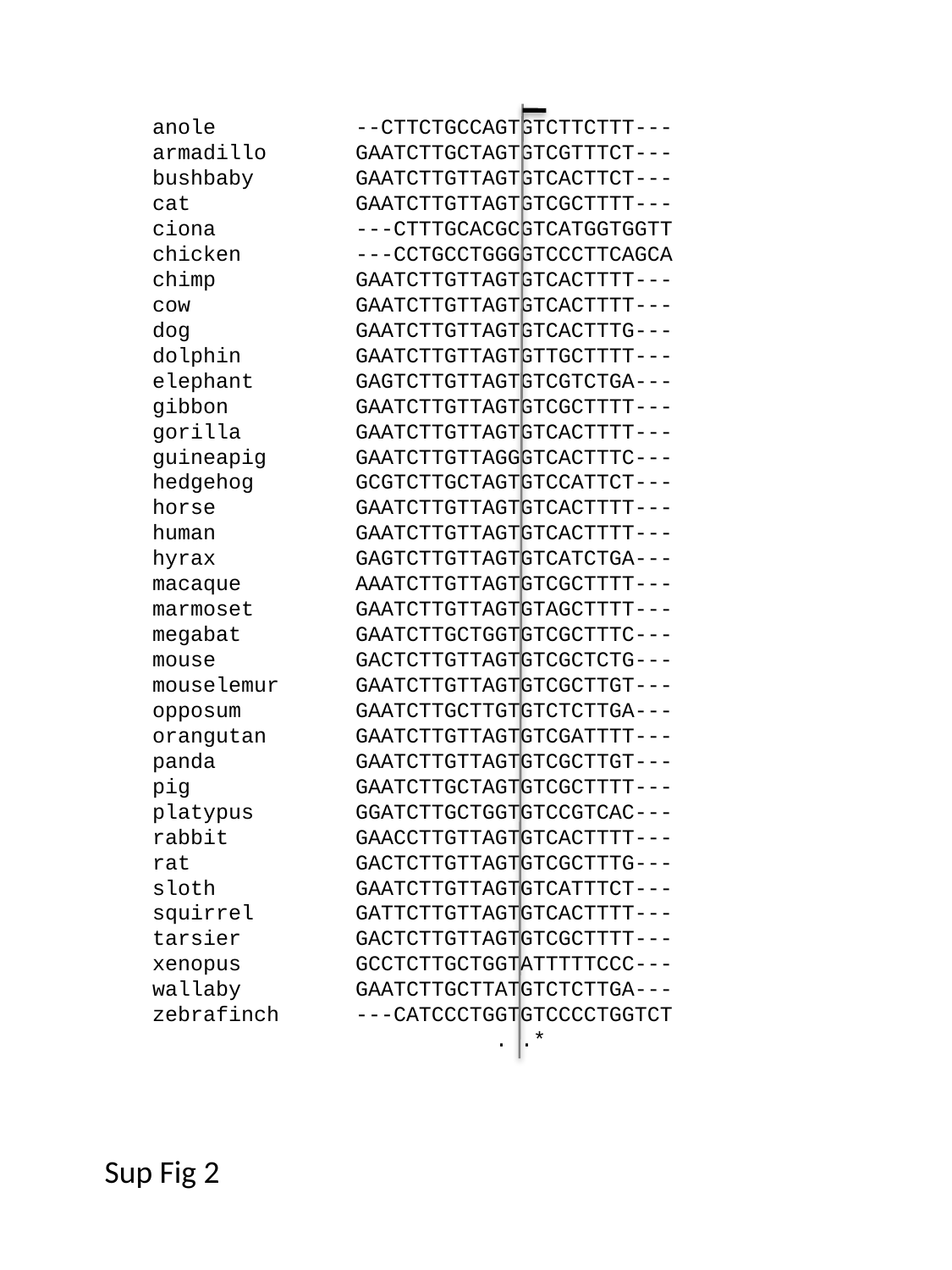

anole --CTTCTGCCAGTGTCTTCTTT---
armadillo GAATCTTGCTAGTGTCGTTTCT---
bushbaby GAATCTTGTTAGTGTCACTTCT---
cat GAATCTTGTTAGTGTCGCTTTT---
ciona ---CTTTGCACGCGTCATGGTGGTT
chicken ---CCTGCCTGGGGTCCCTTCAGCA
chimp GAATCTTGTTAGTGTCACTTTT---
cow GAATCTTGTTAGTGTCACTTTT---
dog GAATCTTGTTAGTGTCACTTTG---
dolphin GAATCTTGTTAGTGTTGCTTTT---
elephant GAGTCTTGTTAGTGTCGTCTGA---
gibbon GAATCTTGTTAGTGTCGCTTTT---
gorilla GAATCTTGTTAGTGTCACTTTT---
guineapig GAATCTTGTTAGGGTCACTTTC---
hedgehog GCGTCTTGCTAGTGTCCATTCT---
horse GAATCTTGTTAGTGTCACTTTT---
human GAATCTTGTTAGTGTCACTTTT---
hyrax GAGTCTTGTTAGTGTCATCTGA---
macaque AAATCTTGTTAGTGTCGCTTTT---
marmoset GAATCTTGTTAGTGTAGCTTTT---
megabat GAATCTTGCTGGTGTCGCTTTC---
mouse GACTCTTGTTAGTGTCGCTCTG---
mouselemur GAATCTTGTTAGTGTCGCTTGT---
opposum GAATCTTGCTTGTGTCTCTTGA---
orangutan GAATCTTGTTAGTGTCGATTTT---
panda GAATCTTGTTAGTGTCGCTTGT---
pig GAATCTTGCTAGTGTCGCTTTT---
platypus GGATCTTGCTGGTGTCCGTCAC---
rabbit GAACCTTGTTAGTGTCACTTTT---
rat GACTCTTGTTAGTGTCGCTTTG---
sloth GAATCTTGTTAGTGTCATTTCT---
squirrel GATTCTTGTTAGTGTCACTTTT---
tarsier GACTCTTGTTAGTGTCGCTTTT---
xenopus GCCTCTTGCTGGTATTTTTCCC---
wallaby GAATCTTGCTTATGTCTCTTGA---
zebrafinch ---CATCCCTGGTGTCCCCTGGTCT
 . .*
Sup Fig 2
