# Supplementary figures and images for: Alternative Transcripts and 3′UTR Elements Govern the Incorporation of Selenocysteine into Selenoprotein S
Source: PLoS One. 2013 Apr 16;8(4):e62102. doi: 10.1371/journal.pone.0062102 (PMC3628699; doi:10.1371/journal.pone.0062102)

## Slide 1
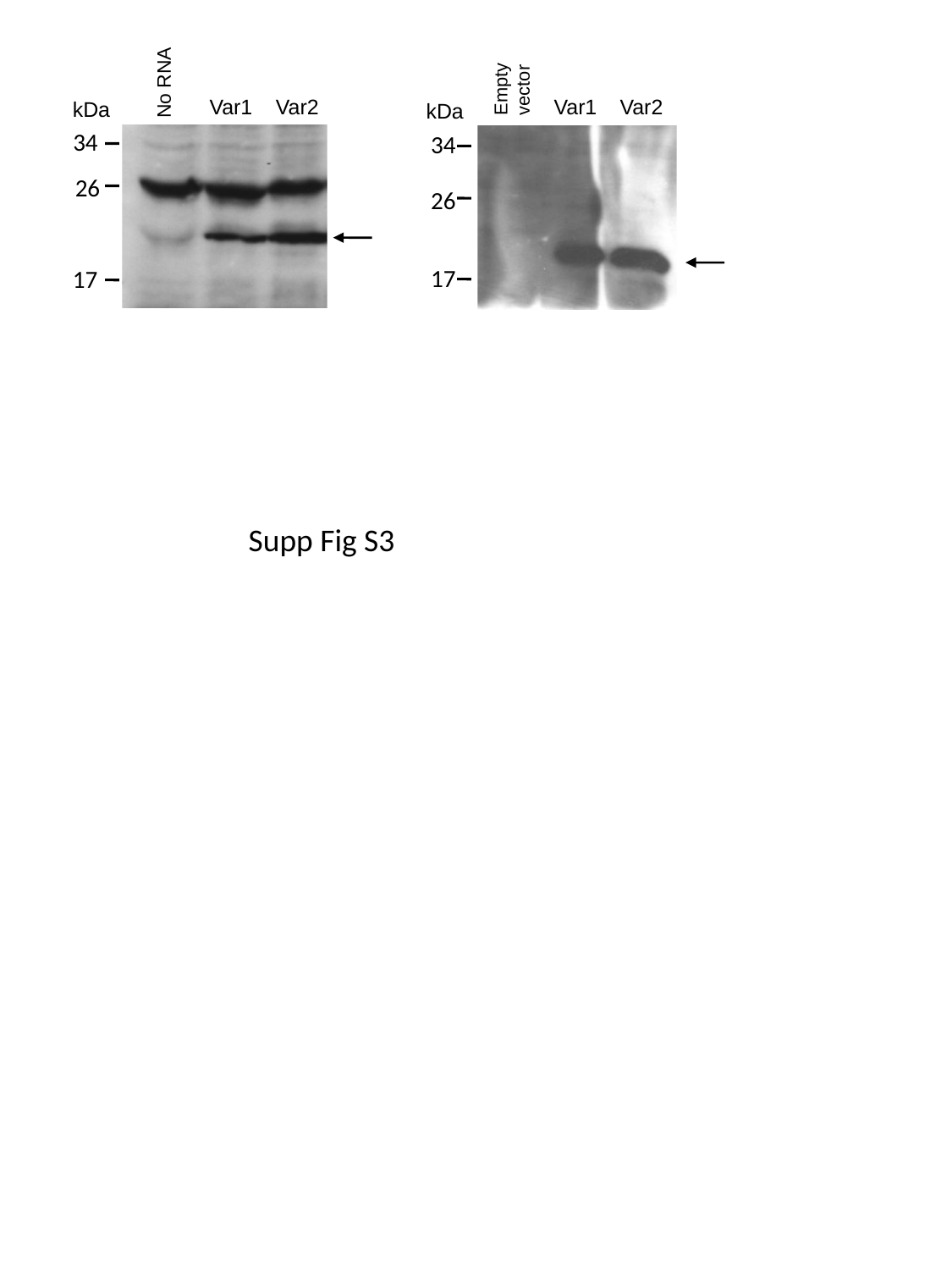

Empty
vector
No RNA
Var1
Var2
Var1
Var2
kDa
kDa
34
34
26
26
17
17
Supp Fig S3

Supplement: Figure S3 — Full-length and truncated SelS proteins are indistinguishable by size. Western blot analysis comparing SelS proteins expressed from variant 1 and variant 2 mRNAs was performed using the α-SelS Prestige antibody (Sigma). A, In vitro translation reactions in RRL were programmed with in vitro transcribed RNAs for SelS-v1 or SelS-v2. A reaction without added RNA was used as a control. B, Transient transfection in HEK293 cells of empty vector (pcDNA3.1), SelS-v1 or Sel-v2. Arrows indicate the SelS protein products. (PPTX) [file pone.0062102.s003.pptx]

## Slide 1
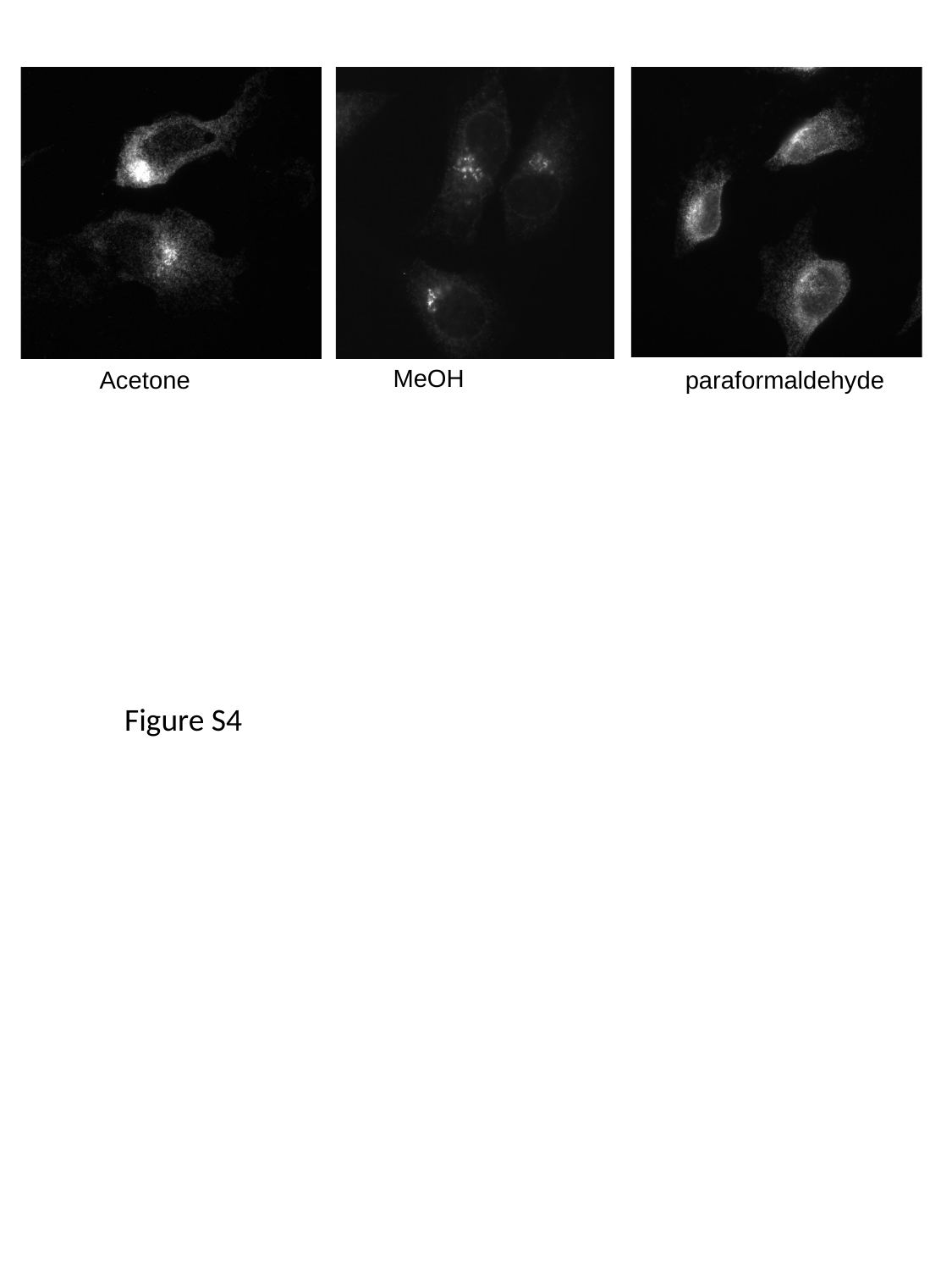

MeOH
Acetone
paraformaldehyde
Figure S4

Supplement: Figure S4 — The perinuclear staining of SelS is not an artifact of the fixation method. U251 cells were fixed either by cold acetone for 5 minutes at −20°C, cold methanol for 5 minutes at −20°C, or 4% paraformaldehyde for 15 minutes at room temperature and the effect on SelS localization was compared. (PPTX) [file pone.0062102.s004.pptx]
