# Supplementary material for: Alternative Transcripts and 3′UTR Elements Govern the Incorporation of Selenocysteine into Selenoprotein S
Source: PLoS One. 2013 Apr 16;8(4):e62102. doi: 10.1371/journal.pone.0062102 (PMC3628699; doi:10.1371/journal.pone.0062102)
Supplement: Table S1 — List of all accession numbers for the SECIS-containing mRNA sequences and the corresponding protein sequences used in this study. ENS-Ensembl database, NM,XM,NP,XP-Genbank database. (DOCX) [file pone.0062102.s005.docx]

| **Common Name** | **Scientific Name** | **RNA accession** | **Protein accession** |
| --- | --- | --- | --- |
| Armadillo | Dasypus novemcincuts | ENSDNOT00000010680 | ENSDNOP00000008278.2 |
| Bushbaby | Otolemur Garnettii | XM_003788628.1 | XP_003788676.1 |
| Cat | Felis catus | ENSFCAT00000029655 | ENSFCAP00000023286 |
| Chicken | Galllus gallus | NM_001024734 | NP_001019905.1 |
| Chimpanzee | Pan troglodytes | NM_001114756.1 | NP_001108228.1 |
| Cow | Bos taurus | NM_001046114.2 | NP_001039579.2 |
| Dog | Canis lupus familiaris | NM_001114757 | NP_001108229.1 |
| Dolphin | Tursiops truncatus | ENSTTRT00000011257 | ENSTTRP00000010673 |
| Elephant | Loxodonta africana | ENSLAFT00000004169 | ENSLAFP00000003476 |
| Frog | Xenopus tropicalis | NM_001011476.1 | NP_001011476.2 |
| Gibbon | Nomascus leucogenys | XM_003281583.1 | XP_003281631 |
| Gorilla | Gorilla gorilla | ENSGGOT00000009000 | ENSGGOP00000008761 |
| Guinea pig | Cavia porcellus | ENSCPOT00000025377 | ENSCPOP00000017370 |
| Hedgehog | Erinaceus europaeus | ENSEEUT00000004396 | ENSEEUP00000003999 |
| Horse | Equus caballus | ENSECAT00000012906 | ENSECAP000000010202 |
| Human | Homo sapiens | NM_018445.4 | NP_060915 |
| Hyrax | Procavia capensis | ENSPCAT00000007799 | ENSPCAP00000007301 |
| Macaque | Macaca mulatta | NM_001114755.1 | NP_001108227.1 |
| Marmoset | Callithrix jacchus | NM_001199926.1 | NP_001186855.1 |
| Megabat | Pteropus vampyrus | ENSPVAT00000012871 | ENSPVAP00000012138 |
| Mouse | Mus musculus | NM_024439.3 | NP_077759.3 |
| Mouse lemur | Microcebus murinus | ENSMICT00000012981 | ENSMICP00000011829 |
| Opossum | Monodelphis domestica | ENSMODG00000012032 | ENSMODP00000015072 |
| Orangutan | Pongo abelii | NM_001200005.1 | NP_001186934 |
| Panda | Ailluropoda melanoleuca | ENSAMET00000007807 | ENSAMEP00000007492 |
| Pig | Sus scrofa | NM_001164113 | NP_001157585 |
| Platypus | Ornithorhynchus anatinus | ENSOANT00000003593 | ENSOANP00000003592 |
| Rabbit | Oryctolagus cuniculus | ENSOCUT00000025451 | ENSOCUP00000017852 |
| Rat | Rattus norvegicus | NM_173120.2 | NP_775143 |
| Sea squirt | Ciona Intestinalis | NM_001190315 | NP_001177244 |
| Sloth | Choloepus hoffmanni | ENSCHOT00000006369 | ENSCHOP00000005624 |
| Squirrel | Spermophilus tridecemlineatus | ENSSTOT00000009035 | ENSSTOP00000008097 |
| Tarsier | Tarsius syrichta | ENSTSYT00000010055 | ENSTSYP00000009223 |
| Tasmanian Devil | Sarcophilus harrisii | ENSSHAT00000007118 | ENSSHAP00000007056 |
| Wallaby | Macropus eugenii | ENSMEUT00000015354 | ENSMEUP00000013974 |
| Zebra Finch | Taeniopygia guttata | NM_001199750.1 | NP_001186679 |
